# Registered report: Cognitive ability, but not cognitive reflection, predicts expressing greater political animosity and favouritism

**DOI:** 10.1111/bjso.12814

**Published:** 2024-11-19

**Authors:** Abigail L. Cassario, Shree Vallabha, Jordan L. Thompson, Alejandro Carrillo, Prachi Solanki, Samantha A. Gnall, Sada Rice, Geoffrey A. Wetherell, Mark J. Brandt

**Affiliations:** ^1^ Department of Psychology Michigan State University East Lansing Michigan USA; ^2^ Department of Psychology Florida Atlantic University Boca Raton Florida USA

**Keywords:** cognitive ability, cognitive reflection, political animosity, worldview conflict

## Abstract

Liberals and conservatives both express political animosity and favouritism. However, less is known about whether the same or different factors contribute to this phenomenon among liberals and conservatives. We test three different relationships that could emerge among cognitive ability, cognitive reflection and political group‐based attitudes. Analysing two nationally representative surveys of US Americans (*N* = 9035) containing a measure of cognitive ability, we find evidence that compared to people lower in cognitive ability, people higher in cognitive ability express *more* animosity towards ideologically discordant groups and more favouritism towards ideologically concordant groups. This pattern was particularly pronounced among liberals. In a registered report study, we then test whether the same is true of cognitive reflection in another large dataset (*N* = 3498). In contrast to cognitive ability, we find no relationship between cognitive reflection, political animosity and favouritism. Together, these studies provide a comprehensive test of how cognitive ability and cognitive reflection are related to political animosity and favouritism for liberals and conservatives in the United States.

## INTRODUCTION

People express negative attitudes towards political outgroups while favouring political ingroups (e.g. Finkel et al., 2020; Iyengar et al., 2019; Mosleh et al., 2021). Despite their differences in policies, values and personalities (e.g. Graham et al., 2009; Sibley & Duckitt, 2008), liberals and conservatives share this tendency to express political animosity and favouritism (Iyengar & Westwood, 2015; Mosleh et al., 2021), although it remains debated whether they express it to the same degree (e.g. Ganzach & Schul, 2021; Stern & Crawford, 2021). This pattern is broadly consistent with the worldview conflict hypothesis, which suggests that both liberals and conservatives dislike ideologically discordant groups and favour ideologically concordant groups (Brandt & Crawford, 2020). In short, groups, in general, are seen either as threatening or supporting one's ideological interests (Brandt et al., 2014; Crawford & Pilanski, 2014; Czarnek et al., 2019; Kossowska et al., 2017; Wetherell et al., 2013) and merely perceiving a group as an ideological ally or opponent is sufficient for the expression of some degree of animosity or favouritism (Brandt & Crawford, 2020; Crawford & Pilanski, 2014; Wetherell et al., 2013).

Most research on political group‐based animosity and favouritism has focused on whether conservatives express more of it than liberals (e.g. Brandt & Crawford, 2020; Ganzach & Schul, 2021; Stern & Crawford, 2021). While an important question, this is not our primary focus. Instead, we start with the premise that both liberals and conservatives express *some degree* of political group‐based animosity and favouritism. We then ask whether cognitive characteristics contribute to animosity and favouritism in the same way for liberals and conservatives, or if cognitive characteristics have different associations with animosity and favouritism depending on people's ideological identities.

We investigate cognitive reflection and cognitive ability because they have been linked with group‐based animosity, particularly among conservatives (e.g. Blanchar & Sparkman, 2020; Onraet et al., 2015). Although these cognitive factors may be relevant to liberals' political group‐based attitudes too, their influence remains largely unexplored. We take up this task and explore how cognitive reflection and cognitive ability predict intergroup attitudes among both liberals and conservatives. We test three perspectives predicting different relationship patterns among cognitive reflection, cognitive ability, ideology and political group‐based attitudes. We test these perspectives across three studies. The first two studies take advantage of existing nationally representative datasets of US Americans where *cognitive ability* and political group‐based attitudes were measured. Then, in the third study, the relationship between cognitive *reflection* and political group‐based attitudes was tested. The third study uses another large existing dataset we did not have access to until in‐principal acceptance of our Stage 1 registered report.

### Cognitive reflection and cognitive ability

Cognitive ability and cognitive reflection are two factors that contribute to people's reasoning. Cognitive ability is an individual's capability to perform higher‐order mental tasks such as problem‐solving, reasoning, remembering and understanding (Onraet et al., 2015). Cognitive reflection is the tendency to override an intuitive but incorrect response in favour of deeper processing (Toplak et al., 2014). The distinction between these two definitions is evident in the constructs' measurements. To score highly on measures of cognitive *ability*, people need to possess the skillset necessary to solve difficult problems. In contrast, to score highly on measures of cognitive *reflection*, people need to expend the mental effort to override an intuitive response and engage in deeper consideration of simple problems.

Despite their differences, the two constructs are empirically related (Pennycook et al., 2015), with recent meta‐analyses showing correlations of about .5 (Otero et al., 2022). These similarities are also apparent in the construct's measurement. To score highly on measures of cognitive *reflection*, people must have some degree of quantitative *ability*. Solving any math problem requires some degree of cognitive ability. Thus, quantitative ability is necessary to score highly on a measure that consists of math problems such as the cognitive reflection test (CRT; Frederick, 2005). Similarly, reflecting on one's conclusions and recognizing that an initial conclusion may be incorrect require the ability to carry out a higher‐order cognitive task.

Critically for our purposes, both constructs predict intergroup attitudes. High cognitive ability relates to more positive attitudes towards low‐status outgroups and more negative attitudes towards high‐status outgroups (Brandt & Crawford, 2016; Hodson & Busseri, 2012; Wodtke, 2016). Likewise, people with lower cognitive reflection and ability express more negative stereotypes about low‐status outgroups than people with higher cognitive reflection and ability (Blanchar & Sparkman, 2020).

The relationship between these two cognitive characteristics and intergroup attitudes suggests that they may be useful in understanding attitudes towards ideological groups. However, the relationship between these constructs is not straightforward, and the relationship between them and animosity may differ. For instance, people higher in cognitive reflection may be better able to monitor and suppress their group‐based attitudes, or alternatively, people higher in cognitive ability may be better able to acquire political information and understand political alliances between groups in society. The ability to recognize ideological alliances and conflicts between groups may lead to higher cognitive ability of individuals to express more political animosity and favouritism. Therefore, we consider both cognitive reflection *and* cognitive ability in our investigation of the relationship between cognitive characteristics and the intergroup attitudes of liberals and conservatives. We test three perspectives that suggest different relationship patterns among cognitive factors, ideology and intergroup attitudes.

### Perspective 1: High cognitive ability and reflection increase tolerance

Working from the premise that a person who dislikes one outgroup is also likely to dislike other outgroups (e.g. Adorno et al., 1950; Allport, 1954; Hodson & Busseri, 2012), scholars have identified personality traits and individual differences that predict group‐based animosity (e.g. Ekehammar & Akrami, 2003; Flynn, 2005; Sibley & Duckitt, 2008). Cognitive ability and cognitive reflection are two such constructs (Blanchar & Sparkman, 2020; Crandall & Eshleman, 2003; Hodson & Busseri, 2012; Wodtke, 2016). Lower levels of both have been linked to negative attitudes towards outgroups (Blanchar & Sparkman, 2020; Hodson & Busseri, 2012; Wodtke, 2016).

Proposed mechanisms behind the relationship vary, but one argument is that monitoring one's prejudices is a cognitively demanding task (Crandall & Eshleman, 2003). Thus, people with lower levels of cognitive ability have been suggested to express more animosity than those higher in the construct (Crandall & Eshleman, 2003). This hypothesis was put forth prior to the emergence of the cognitive reflection construct (Frederick, 2005). Cognitive reflection is directly linked to the ability to monitor and suppress intuitive but incorrect responses in favour of deliberation (Saribay & Yilmaz, 2017; Toplak et al., 2014; Yilmaz & Saribay, 2017). This suggests that the mechanism put forth by Crandall and Eshleman (2003) should extend to cognitive reflection as well.

Consistent with these ideas, people with lower levels of verbal ability and cognitive reflection express more prejudicial racial attitudes and racial and ethnic stereotypes than people with higher levels of verbal ability and cognitive reflection (e.g. Blanchar & Sparkman, 2020; Wodtke, 2016). Others have uncovered a similar relationship (Hodson & Busseri, 2012), but have suggested a slightly different mechanism. They suggest that higher cognitive ability should relate to lower intergroup animosity for two reasons (Hodson & Busseri, 2012). First, greater cognitive capacity allows people to better adopt the perspectives of outgroup members. Second, people higher in cognitive ability are less likely to adopt intolerant ideologies. In line with both perspectives, those who are *lower* in cognitive ability and cognitive reflection should express *more* animosity towards groups broadly speaking than people higher in cognitive ability and cognitive reflection, who should be generally tolerant (H1a).

Others have noted that people lower in cognitive ability and cognitive reflection may be drawn to conservative ideologies (e.g. Eidelman et al., 2012; Stankov, 2009). This is because these ideologies are characterized by ideas and policies that condone animosity towards groups and entrench inequality in society (e.g. Jost, 2006, 2017; Jost et al., 2003). Some go as far as to suggest that the relationship between cognitive factors and intergroup attitudes is *mediated* through the adoption of conservative ideologies that condone, and at times encourage, such animosity (e.g. Hodson & Busseri, 2012; Meisenberg, 2015). Therefore, this suggests that the effect of cognitive reflection and cognitive ability on intergroup attitudes predicted by H1a should weaken once ideology is accounted for; liberals on average should be inclined towards tolerance, while conservatives on average should be inclined towards animosity (H1b).

### Perspective 2: Cognitive ability and reflection increase political animosity and favouritism among liberals, but decrease it among conservatives

While it is possible that higher levels of cognitive ability and cognitive reflection lead to tolerance, it is also possible that these cognitive characteristics *differentially* predict political animosity and favouritism depending on respondent's political ideology. This is because both respondent ideology *and* cognitive ability have been shown to contribute to the development of animosity and favouritism towards the same set of target groups. Thus, it is possible that these constructs exert influence on intergroup attitudes in a way that either reinforces or counteracts the other's influence.

This perspective is derived from studies testing the relationship between ideology and cognitive ability with intergroup attitudes. Research examining the relationship between cognitive ability and attitudes towards diverse, ideologically varied groups suggests that individuals *high* in cognitive ability tend to favour liberal groups. In contrast, these individuals disfavour conservative groups. Individuals *low* in cognitive ability tend to favour conservative groups while disfavouring liberal groups (Brandt & Crawford, 2016; De Keersmaecker et al., 2021).

The relationship between a person's political ideology and animosity is also moderated by target group ideology (e.g. Brandt, 2017; Brandt et al., 2014). Specifically, liberals favour groups that are liberal, while disfavouring groups that are conservative. Conservatives display exactly the opposite pattern, favouring groups that are conservative while disfavouring groups that are liberal (Brandt, 2017).

Taken together, the evidence from this work suggests that high cognitive ability and liberal ideology predict animosity towards similar groups, and low cognitive ability and conservative ideology predict animosity towards similar groups. As such, this framework suggests that a significant three‐way interaction effect among respondent's ideology, respondent's cognitive ability and the ideology of the group being judged might emerge. Specifically, among conservatives, high cognitive ability should lead target group ideology to be *less* predictive of animosity and favouritism than among conservatives with low cognitive ability. This is because high cognitive ability and conservative ideology influence the effect of group ideology on attitudes in opposing directions (i.e. they may cancel each other out). High cognitive ability is associated with greater animosity towards conservative groups while conservative ideology is associated with greater animosity towards liberal groups. However, for lower cognitive ability conservatives, the opposite pattern should emerge. In this case, low cognitive ability and conservative ideology reinforce the influence of target group ideology on attitudes (H2a), as both low cognitive ability and conservative ideology are associated with higher animosity towards liberal groups (i.e. the effect of target group ideology on attitudes should be stronger among low cognitive ability conservatives than among high cognitive ability conservatives).

Among liberals high in cognitive ability, liberal ideology and high cognitive ability predict animosity towards similar groups and therefore should reinforce the influence of target group ideology on attitudes. Thus, among high cognitive ability liberals, the effect of target group ideology on political animosity and favouritism should be greater than among liberals lower in cognitive ability. In contrast, when considering liberals low in cognitive ability, ability and ideology should exert competing influences on the effect of target group ideology on attitudes. Liberals lower in cognitive ability should thus express less favouritism towards liberal groups and animosity towards conservative groups than liberals higher in cognitive ability (H2b) (i.e. the effect of target group ideology on attitudes should be stronger among high cognitive ability liberals than among low cognitive ability liberals). In short, H2 predicts that higher cognitive ability and reflection should predict greater political animosity and favouritism among liberals, but less among conservatives.

### Perspective 3: Cognitive ability and reflection increase political animosity and favouritism

Both liberals and conservatives express some degree of animosity towards ideologically discordant groups, or in other words, groups that do not share their values (Brandt et al., 2014; Brandt & Crawford, 2020). A critical pre‐requisite for value conflict to shape attitudes is that perceivers *recognize* such value conflict, to begin with. People higher in cognitive ability and cognitive reflection may be better at recognizing value conflict, especially in the political domain, than those lower in these constructs.

To recognize value conflict, people must first be able to recognize the contours of socio‐political debates. Notably, people are typically low in political knowledge (Carpini & Keeter, 1993, 1996) and unable to correctly characterize ideological divides (Converse, 1964; Kinder & Kalmoe, 2017). However, people higher in cognitive ability and cognitive reflection may be better able to acquire the requisite political knowledge to recognize political conflict, and associate groups with ideological positions. Cognitive ability might contribute to political knowledge as the cognitive capacity to process information, store it in memory and connect it to other information is important for knowledge acquisition (Carpini & Keeter, 1996). To acquire political knowledge, individuals must also spend time and energy reflecting on the (sometimes complex) political information they encounter (Carpini & Keeter, 1996). Accordingly, people with higher levels of cognitive ability and reflection may be better able to recognize the ideological orientations of groups and link the information with their own ideological interests.

This reasoning is consistent with several different findings. For example, it is consistent with the finding that political extremity is more strongly related to outgroup animosity for people higher in cognitive ability (Ganzach & Schul, 2021). It is also consistent with the finding that the possession of greater cognitive skills and education is correlated with greater attachment to political parties, more political knowledge and more coherent political ideologies (Albright, 2009; Barabas et al., 2014; Carpini & Keeter, 1993; Converse, 1964). Similarly, some work suggests that people higher in cognitive reflection are more likely to reach politically biased conclusions (e.g. Kahan, 2013). Likewise, it aligns with the notion that people higher in cognitive reflection are more likely to recognize ideological divisions and hold strong priors supporting their own side's agenda (Pennycook & Rand, 2019; Tappin et al., 2020, 2021). In other words, people who are more reflective and have greater cognitive ability could be better able to acquire, defend and rationalize political animosities (e.g. Lick et al., 2018). As such, this perspective suggests that people with higher levels of cognitive ability and reflection should be more likely to possess the knowledge necessary to characterize ideological conflict between politically relevant groups. Thus, this perspective suggests that people higher in cognitive reflection and cognitive ability should be more likely to express animosity towards ideologically discordant groups, and favouritism towards ideologically concordant groups (H3).

### The current research

We investigate how cognitive ability and cognitive reflection are associated with animosity and favouritism towards politically relevant groups among liberals and conservatives using three differing perspectives. The first perspective suggests that individuals higher in cognitive ability and reflection will express less negative attitudes than those lower in these constructs, but that the relationship should weaken once ideology is accounted for. The second perspective predicts that higher cognitive ability and reflection should predict greater political animosity towards ideologically discordant groups and favouritism towards ideologically concordant groups among liberals, but less animosity towards ideologically discordant groups and favouritism towards ideologically concordant groups among conservatives. The third perspective predicts that people higher in cognitive ability and cognitive reflection should express more animosity towards ideologically discordant groups and more favouritism towards ideologically concordant groups than people lower in cognitive ability and cognitive reflection.

We first test these perspectives in two nationally representative samples. In the 2012 and 2016 waves of the American National Election Study (ANES), participants completed the WORDSUM task, a proxy measure of cognitive ability as well as measures of political ideology and explicit attitudes towards a variety of groups. These groups include political groups (e.g. liberals), religious groups (e.g. Christian fundamentalists), activist groups (e.g. feminists) and identity‐based groups (e.g. women). These data were merged with ratings of group ideology (Brandt, 2017; Brandt & Crawford, 2016) collected from separate samples of Americans. This provides us with the necessary information to determine the extent a target group is generally seen as politically concordant or discordant with the participant. Studies 1 and 2 (the 2012 and 2016 waves of the ANES) are discussed together as they contain many of the same measures and use a similar sampling procedure. Then, we discuss our registered report study. The registered report study consists of a pre‐registered analysis of the Ideology 2.0 dataset (Schmidt et al., 2022). These data were collected from the Project Implicit website and were only made available to us after our Stage 1 registered report received an in‐principle acceptance. Importantly, the Ideology 2.0 dataset contains a measure of cognitive reflection rather than cognitive ability, and relative rather than absolute measures of political animosity and favouritism.

## METHOD: STUDIES 1 AND 2

Our individual‐level data for Studies 1 and 2 come from the 2012 and 2016 waves of the nationally representative ANES (2012: *N* = 5783, *M*_age_ = 49.62, *SD*_age_ = 16.85, 2783 men and 2985 women; 2016: *N* = 4122, *M*_age_ = 49.58, *SD*_age_ = 17.58, 1937 men, 2174 women and 11 other gender identity). The surveys used both face‐to‐face interviews and computer‐assisted questionnaires. We control for survey mode in all reported analyses. In Table 1, we give a summary of the measures we use in testing our hypotheses in Studies 1 and 2.

**TABLE 1:** Summary of Study 1 and Study 2 measures.

Measure	*N* items	Description
Cognitive Ability	10	Measured with WORDSUM. A 10‐item vocabulary test (Thorndike, 1942). Participants were asked to select which of five words best matched the meaning of a target word. Mean scores were rescaled to range from 0 to 1 and mean centred for analysis. The measure is strongly correlated with general intelligence (Wolfle, 1980) and verbal ability (Kan et al., 2013). It is a commonly used measure of cognitive ability (e.g. Brandt & Crawford, 2016; Malhotra et al., 2007). It is also significantly correlated with conceptually related variables such as years of education, parents' educational attainment and IQ in childhood (see Wolfle, 1980)
Group‐Based Animosity/Favouritism	1/Group	Assessed with feeling thermometer ratings towards 24 groups in society in 2012 and 21 groups in 2016. Feeling thermometers were rescaled to range from 0 (cold/unfavourable) to 100 (warm/favourable). For these analyses, we reverse‐scored items such that higher scores indicated more political animosity and lower scores indicated group‐based favouritism. For ease of communication, this measure is referred to as capturing political animosity when describing results. Scores were rescaled to range from 0 to 1 for purposes of coefficient interpretation. Page 3 in the Supporting Information includes specific target groups in the 2012 and 2016 waves ANES
Ideology	1	Political ideology was measured using a 7‐point Likert scale ranging from 1 (extremely liberal) to 7 (extremely conservative) and was centred at the scale midpoint (i.e. ‘Moderate; middle of the road’). Participants who reported ‘don't know’ and ‘haven't thought about it much’ were excluded from analyses, but results are substantively unchanged when they are included (coded at the midpoint)
Demographics	5	We control for demographics, including race (contrasts = White people vs. non‐White people, Black people vs. people who belong to other minority groups, and Hispanic people vs. other minority group members except Black people), gender (dummy coded and mean centred, male = 1), income, education and age. Age, education and income are rescaled to range from 0 to 1 and were mean centred. Results are reported without control variables in Tables SA – *SD* in the Supporting Information. Our primary results are consistent regardless of the inclusion of covariates
Group Ideology Ratings	1/Group	Group ideology ratings for the 2012 and 2016 ANES studies come from previous studies that used Amazon's Mechanical Turk participants who resided in the United States (2012 groups: Brandt & Crawford, 2016; 2016 groups: Brandt, 2017). In both studies, people rated the groups on several dimensions, including perceived ideology. The perceived ideology measure ranged from 0 to 100 with higher ratings indicating that a group was perceived as more conservative (see Brandt, 2017, and Brandt & Crawford, 2016, for complete study details). Perceived ideology of a group helps us identify the extent groups are seen as consistent or inconsistent with a participant's own ideological orientations (e.g. Brandt, 2017). The intraclass correlation (ICC) of the group ideology rating was 0.99, showing a high degree of consensus in perceived ideology (see also Koch et al., 2020). Group ideology ratings for both studies were recoded to range from 0 to 1 and midpoint centred

### Modelling strategy

We estimated multilevel models with group attitudes nested within participants. We included random intercepts for target group and participant. Target group ideology was included as a random slope. Our rescaling of variables (to range from 0 to 1) means that regression coefficients for the main effects represent the expected change in the dependent variable upon moving from the minimum (0) to the maximum (1) of the respective independent variable. Markov chain Monte Carlo (MCMC) power analyses conducted in the simr package (Green & MacLeod, 2016) suggest we possess approximately 100% power to detect a small three‐way interaction (*b* = .44) in both the 2012 and 2016 datasets. We also report standardized β coefficients, where variables are standardized by standard deviation.

## RESULTS: STUDIES 1 AND 2

We tested hypotheses 1a‐3. The hypotheses, models and pattern of results they correspond to are in Table 2 below.

**TABLE 2:** Hypotheses, models and predicted pattern of results for Perspectives 1–3 in Studies 1 and 2.

Hypothesis	Model	Terms of interest/predicted pattern of results
H1a: Individuals higher in cognitive ability are more tolerant (without accounting for respondent ideology)	Main effects model. DV: Feeling thermometer ratings towards groups. IVs: Group ideology, cognitive ability and demographic control variables	Cognitive ability: Negative relationship suggesting that higher cognitive ability generally corresponds to less negative attitudes towards groups
H1b: After accounting for respondent ideology, the effect of cognitive ability on attitudes should reduce in magnitude	Main effects model. DV: Feeling thermometer ratings towards groups. IVs: Group ideology, cognitive ability, *respondent ideology* and demographic control variables	Cognitive ability: Smaller negative relationship compared to model for H1a Respondent ideology: The variable is recoded such that higher values represent more conservative ideology. Thus, there should be a positive relationship, with more conservative individuals expressing more animosity
H2a: Among conservatives, higher levels of cognitive ability should predict less political animosity (i.e. the effect of target group ideology on attitudes towards ideological groups should be weaker for high cognitive ability conservatives than low cognitive ability conservatives)	Model with a three‐way interaction. DV: Feeling thermometer ratings towards groups. IVs: group ideology, respondent ideology, cognitive ability and demographic control variables. Two‐way interactions between group ideology and cognitive ability, group ideology and respondent ideology and respondent ideology and cognitive ability. *Three‐way interaction among respondent ideology, group ideology and cognitive ability* Simple slopes analysis for the three‐way interaction term. Examines the effect of group ideology on political animosity among high and low cognitive ability liberals and conservatives	Three‐way interaction: negative relationship indicating ability is more predictive of animosity among liberals than conservatives Simple slopes analysis: the absolute value of the coefficient for group ideology on attitudes towards ideological groups should be *smaller* for high cognitive ability conservatives than for lower cognitive ability conservatives. This pattern signals they express *less* political animosity than lower‐ability conservatives do
H2b: Among liberals, higher cognitive ability should predict more political animosity towards ideologically discordant groups. (i.e. The effect of target group ideology on attitudes towards ideological groups should be stronger for high cognitive ability liberals than low cognitive ability liberals.)	Same model as H2a Simple slopes analysis for the three‐way interaction term. Examines the effect of group ideology on political animosity among high and low cognitive ability liberals and conservatives	Three‐way interaction: Negative relationship indicating ability is more predictive of animosity among liberals than conservatives Simple slopes analysis: absolute value of the coefficient for group ideology on attitudes towards ideological groups should be *larger* among high cognitive ability liberals than among lower cognitive ability liberals. This pattern signals they express *more* political animosity towards ideologically discordant groups than lower‐ability liberals do
H3: Among both liberals and conservatives, high cognitive ability should predict greater animosity towards ideologically discordant groups	Same model as H2 Simple slopes analysis for the three‐way interaction term. Examines the effect of target group ideology on animosity across levels of respondent ideology and cognitive ability	Simple slopes analysis: Absolute value of the coefficients for group ideology should be *larger* among high cognitive ability liberals *and* conservatives than among low cognitive ability liberals *and* conservatives. This signals that high cognitive ability idealogues express more political group‐based animosity than lower cognitive ability ideologues do

*Note:* Feeling thermometer ratings towards groups are always recoded such that higher ratings correspond to *higher* levels of animosity and lower ratings correspond to higher levels of favouritism.

The first model fitted to test H1a reveals that although the effect of cognitive ability on animosity was negative in 2012, it is very close to zero and far from statistical or substantive significance. In the 2016 model, the coefficient flips directions and is again small and nonsignificant. This is inconsistent with the first perspective which suggests that individuals higher in cognitive ability should be generally tolerant.

The main effects of respondent ideology from the 2012 and 2016 models that were added to the model to test H1b reveal that liberals and conservatives are similarly likely to express animosity. This result is inconsistent with the literature that predicts conservatives should be disposed towards animosity, whereas liberals are disposed towards tolerance. Results from the 2012 model are shown in Table 3, while results of the 2016 model are shown in Table 4.

**TABLE 3:** Fixed effects from main effects models in the 2012 ANES excluding and including respondent ideology.

Variable	2012 ANES			
	Without respondent ideology		With respondent ideology	
	*b* (SE)	β (SE)	*b* (SE)	β (SE)
Survey Mode	.04*** (.03)	.166*** (.13)	.04*** (.03)	.166*** (.01)
White People/Non‐White People	.03*** (.00)	.11*** (.01)	.03*** (.00)	.11*** (.01)
Black People/Other Minority Groups	−.04*** (.01)	−.13*** (.01)	−.04*** (.01)	−.13*** (.02)
Hispanic People/Other Minority Groups Except Black People	−.04*** (.007)	−.16*** (.02)	−.04*** (.007)	−.16*** (.03)
Gender	02*** (.002)	.09*** (.03)	.02*** (.002)	.09*** (.01)
Income	−.009 (.005)	−.009 (.006)	−.009 (.005)	−.001 (.006)
Age	−.07*** (.01)	−.06*** (.005)	−.07*** (.01)	−.06*** (.006)
Ideology of Group	−.07 (.12)	−.05 (.10)	−.07 (.12)	.05 (.10)
Education	−.02** (.006)	−.02** (.01)	−.02** (.01)	−.02** (.006)
*Cognitive Ability*	−.002 (.007)	−.002 (.006)	−.002 (.007)	−.002 (.006)
*Ideology of Respondent*	–	–	.001 (.006)	.001 (.005)

*Note*: **p < .01 and ***p < .001. Note that rounded coefficients are reported in the table, but significance comes from exact results. For continuous variables, standardized β coefficients represent the expected change in standard deviation units in the dependent variable per 1 *SD* unit change in the respective independent variable. For categorical variables, coefficients represent expected standard deviation change in the dependent variable, if a member of a given category.

**TABLE 4:** Fixed effects from main effects models in the 2016 ANES excluding and including respondent ideology.

Variable	2016 ANES			
	Without respondent ideology		With respondent ideology	
	*b* (SE)	β (SE)	*b* (SE)	β (SE)
Survey Mode	.01** (.004)	.04*** (.02)	.01** (.004)	.05** (.02)
White People/Non‐White	.02*** (.00)	.07*** (.02)	.01** (.00)	.06** (.02)
Black People/Other Minority Groups	−.006 (.01)	−.02 (.03)	.003 (.01)	.01 (.03)
Hispanic People/Other Minority Groups Except Black People	−.03*** (.008)	−.10*** (.03)	−.03*** (.01)	−.10*** (.04)
Gender	.03*** (.003)	.11*** (.01)	.03*** (.004)	.12*** (.02)
Income	−.02* (.007)	−.02* (.008)	−.02* (.007)	−.02* (.008)
Age	−.03*** (.01)	−.03*** (.008)	−.03*** (.01)	−.03*** (.008)
Ideology of Group	.03 (.09)	.03 (.09)	.04 (.10)	.04 (.10)
Education	−.04** (.01)	−.03** (.008)	−.03* (.01)	−.02* (.009)
*Cognitive Ability*	.006 (.009)	.005 (.007)	.01 (.01)	.01 (.009)
*Ideology of Respondent*	–	–	−.01 (.01)	−.01 (.008)

*Note:* *p < .05, **p < .01 and ***p < .001. For continuous variables, standardized β coefficients represent the expected change in standard deviation units in the dependent variable per 1 *SD* unit change in the respective independent variable. For categorical variables, coefficients represent expected standard deviation change in the dependent variable if a member of the category.

To replicate previous research (e.g. Brandt et al., 2014) and for the purposes of model building (Gelman & Hill, 2006), we also fit two‐way interaction models. These models fitted in the 2012 and 2016 data include the two‐way interaction terms between group ideology and participant ideology, participant ideology and cognitive ability, and group ideology and cognitive ability. Results show that participant ideology and cognitive ability significantly interacted with target group's ideology. Cognitive ability did not significantly interact with respondent ideology. While these models do not directly relate to the hypotheses posed here, they replicate prior work establishing that liberals and high cognitive ability individuals express animosity towards conservative groups, and favouritism towards liberal groups (e.g. Brandt, 2017; Brandt & Crawford, 2016). Results of the two‐way interaction models are displayed in Table 5.

**TABLE 5:** Fixed effects of two‐way interaction models in the 2012 and 2016 ANES.

Variable	2012 ANES		2016 ANES	
	*b* (SE)	β (SE)	*b* (SE)	β (SE)
Survey Mode	.04*** (.03)	.166** (.01)	.01** (.004)	.05** (.02)
White People/Non‐White	.03*** (.00)	.11*** (.01)	.01*** (.00)	.05** (.02)
Black People/Other Minority Groups	−.04*** (.01)	−.13*** (.02)	.003 (.01)	.01 (.03)
Hispanic People/Other Minority Groups Except Black People	−.04*** (.01)	−.16*** (.03)	−.03*** (.01)	−.10** (.03)
Gender	.02*** (.00)	.09*** (.01)	.03*** (.00)	.12*** (.01)
Income	−.009 (.01)	−.009 (.1)	−.02* (.01)	−.02* (.01)
Age	−.07*** (.01)	−.06*** (.005)	−.03*** (.01)	−.03*** (.01)
Ideology of Group	−.03 (.12)	−.06 (.10)	.06 (.10)	.03 (.10)
Education	−.02*** (.006)	−.02*** (.006)	−.03* (.01)	−.02* (.008)
Cognitive Ability	−.005 (.01)	.001 (.006)	.003 (.01)	.003 (.009)
Ideology of Respondent	.02 (.01)	−.002 (.006)	.05*** (.01)	.06*** (.008)
Ideology of Group*Ideology of Respondent	−1.42*** (.02)	−.28*** (.004)	−1.42*** (.02)	−.36*** (.006)
Cognitive Ability*Ideology of Respondent	−.02 (.02)	−.005 (.005)	−.02 (.03)	−.006 (.008)
Cognitive Ability*Ideology of Group	.23*** (.02)	.04*** (.004)	.25*** (.03)	.06*** (.006)

*Note*: *p < .05; **p < .01 and ***p < .001. Note that coefficients in the table are rounded, but significance is calculated with exact values. Standardized β coefficients for continuous variables represent expected standard deviation change in dependent variable per 1 *SD* unit change in the respective independent variable. Standardized coefficients for categorical variables represent expected standard deviation change in the dependent variable if a member of the respective category.

**FIGURE 1:**
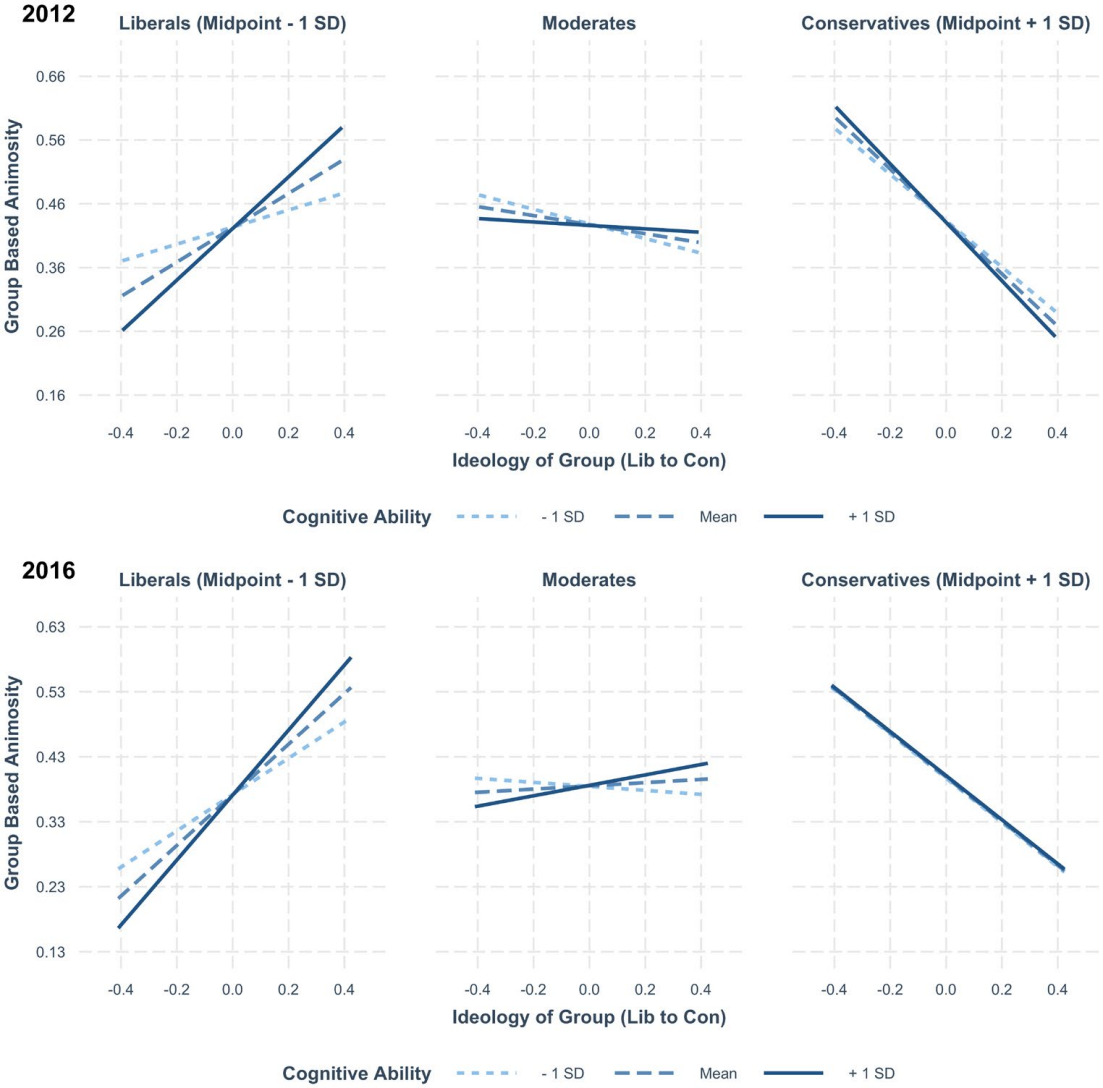
Effect of target group ideology on political animosity and favouritism across high and low cognitive ability liberals, conservatives and moderates. This figure displays the pattern of interaction uncovered in the 2012 and 2016 waves of the ANES. The Y‐axis is scaled at ±1 *SD* of the mean of the DV for both studies (Witt, 2019). Results are largely similar across both waves. Among liberals, individuals higher in cognitive ability express more animosity towards ideologically discordant groups and more favouritism towards ideologically concordant groups. Among conservatives, a similar, though weaker, pattern emerged in 2012, but not in 2016.

These estimates, however, are all qualified by a significant three‐way interaction among participant ideology, target ideology and cognitive ability in our final model. This interaction is key for testing Perspectives 2 and 3. Breaking down the key three‐way interaction, we find that the two‐way interaction between group ideology and cognitive ability is significant among liberals in both studies (2012: *b =* .69, *SE* = .04, β = .13, *p* < .001; 2016: *b* = .58, *SE* = .05, β = .13, *p* < .001) and only significant for conservatives in the 2012 study (2012: *b* = −.21, *SE* = .05, β = −.04, *p* < .001; 2016: *b* = .01, *SE* = .05, β = .002, *p* = .84). This indicates that high cognitive ability liberals *and* conservatives express more political animosity and favouritism in 2012 than low cognitive ability liberals and conservatives do. In 2016, liberals showed the same pattern, but high and low cognitive ability conservatives do not differ in the amount of political animosity and favouritism they express (Table 6). The interaction pattern is displayed in Figure 1 and the simple slopes are shown in Table 7.

**TABLE 6:** Fixed effects of three‐way interaction models in the 2012 and 2016 ANES.

Variable	2012 ANES		2016 ANES	
	*b* (SE)	β (SE)	*b* (SE)	β (SE)
Survey Mode	.04*** (.00)	.17*** (.01)	.01*** (.00)	.05** (.02)
White People/Non‐White	.03*** (.00)	.11** (.01)	.01** (.004)	.05** (.02)
Black People/Other Minority Groups	−.04*** (.01)	−.13*** (.02)	.00 (.01)	.01 (.03)
Hispanic/Other Minority Groups Except Black People	−.04*** (.01)	−.16*** (.03)	−.03*** (.00)	−.10** (.04)
Gender	.02*** (.00)	.09*** (.01)	.03*** (.00)	.12*** (.02)
Income	−.01 (.01)	−.009 (.006)	−.02* (.007)	−.02* (.008)
Age	−.07*** (.01)	−.06*** (.006)	−.03*** (.007)	−.03*** (.008)
Ideology of Group	−.03 (.13)	−.06 (.10)	.05 (.10)	.02 (.10)
Education	−.02*** (.01)	−.02* (.006)	−.03* (.01)	−.02* (.008)
Cognitive Ability	.00 (.01)	−.001 (.006)	.002 (.01)	.003 (.009)
Ideology of Respondent	.02** (.01)	−.002 (.005)	.05*** (.007)	.05*** (.008)
Ideology of Group* Ideology of Respondent	−1.39*** (.02)	−.27*** (.004)	−1.36*** (.02)	−.34*** (.005)
Cognitive Ability*Ideology of Respondent	.00 (.03)	−.006 (.005)	.04 (.04)	.004 (.008)
Cognitive Ability*Ideology of Group	.24*** (.02)	.035*** (.004)	.26*** (.03)	.05*** (.005)
Ideology of Group* Cognitive Ability*Ideology of Participant	−1.63*** (.09)	−.07*** (.004)	−.88*** (.10)	−.05*** (.006)

*Note*: *p < .05, **p < .01 and ***p < .001. Note that coefficients reported in the table are rounded, significance is based on exact results. Standardized β coefficients for continuous variables represent expected standard deviation change in dependent variable per 1 *SD* unit change in the respective independent variable. Standardized coefficients for categorical variables represent expected standard deviation change in the dependent variable if a member of the respective category.

**TABLE 7:** Simple slopes of group ideology on animosity for 2012 and 2016 ANES.

	Liberal participants (midpoint −1 *SD*)			Conservative participants (midpoint +1 *SD*)		
	*b*	SE	β	*b*	SE	β
2012 ANES						
Lower cognitive ability (Mean − 1 *SD*)	.12	.13	.11	−.34**	.13	−.29
Higher cognitive ability (Mean + 1 *SD*)	.38***	.13	.32	−.44***	.13	−.36
2016 ANES						
Lower Cognitive Ability (Mean − 1 *SD*)	.30***	.10	.29	−.34***	.10	−.32
Higher Cognitive Ability (Mean + 1 *SD*)	.51***	.10	.49	−.33***	.10	−.31

*Note*: ** *p* < .01 ***p < .001. β coefficients are standardized by standard deviation units.

The three‐way interaction term is negative and significant, as predicted by Perspective 2; but the overall pattern of results is most consistent with the predictions generated by Perspective 3. This perspective suggests that people with greater cognitive ability and thus tendencies to notice ideological conflict should express more group‐based animosity and favouritism than people lower in cognitive ability. We interpret our results as more consistent with Perspective 3 than Perspective 2 because Perspective 2 predicts that low cognitive ability conservatives should express *more* political animosity and favouritism than high cognitive ability conservatives. Neither study shows evidence of this pattern. Both studies provide support for the premise that high cognitive ability liberals express more political animosity and favouritism than low cognitive ability liberals, and Study 1 shows the same pattern among conservatives. We note, however, that our results are noticeably weaker among conservatives, and encourage further research on the relationship between cognitive ability and political animosity among conservatives.^1^

## DISCUSSION: STUDIES 1 AND 2

The results of Studies 1 and 2 provided evidence most consistent with Perspective 3; that individuals high in cognitive ability express more political animosity and favouritism than those low in cognitive ability. According to this perspective, individuals who are higher in cognitive ability are better able to recognize ideological conflict and determine which groups align or conflict with their own ideological orientations. Support for this premise was consistent among liberals, but mixed among conservatives.

The effects of both participant ideology and cognitive ability were near zero and nonsignificant. When examining attitudes towards a wide variety of target groups, conservatives and people lower in cognitive ability were no more likely to express animosity than liberals and people higher in cognitive ability. This is inconsistent with Perspective 1. Both Perspective 2 and Perspective 3 predict that among liberals, higher cognitive ability should predict greater political group‐based animosity and favouritism. This idea receives empirical support. However, Perspective 2 also predicts that among conservatives, higher cognitive ability should predict expressing *less* political animosity and favouritism. This pattern does not emerge in either study. In 2012, conservatives higher in cognitive ability expressed *more* political animosity and favouritism, in line with Perspective 3, whereas, in 2016, higher cognitive ability conservatives behaved no differently than lower cognitive ability conservatives.

The empirically small difference between the 2012 and 2016 results for conservatives could have emerged for several reasons, including the smaller ANES sample size, the specific set of ideological groups under study, the highly polarized socio‐political context in which data were collected or sampling error.

In Study 3, we shift from testing cognitive ability to testing cognitive reflection. It is possible that reflection functions similarly to cognitive ability. However, cognitive reflection is a distinct construct. By testing cognitive reflection, we can more fully explore how cognitive factors are associated with political group‐based animosity across ideological lines.

## METHOD STUDY 3: IDEOLOGY 2.0 DATASET

### Overview

This study uses a combination of pre‐existing data, including a measure of cognitive reflection (Frederick, 2005) from the Ideology 2.0 dataset (Schmidt et al., 2022), and new group ideology ratings collected for this project. Studies 1 and 2 included a widely accepted measure of group‐based animosity and favouritism, capturing absolute levels of favouritism and animosity. However, work on political animosity (e.g. Finkel et al., 2020; Iyengar & Westwood, 2015; Mosleh et al., 2021) and prejudice more generally (e.g. Bergh & Brandt, 2022; Graziano et al., 2007; Greenwald et al., 1998) often focuses on attitudes towards politically concordant groups *relative* to politically discordant groups. The Ideology 2.0 dataset contains measures of relative, rather than absolute, political group‐based animosity and favouritism, helping us extend our exploration to a new measure of group attitudes. This study thus allows us to build on our previous studies in two important ways. First, we examine the relationship between cognitive *reflection* and political animosity and favouritism. Second, we examine the relationship between cognitive reflection and *relative* political animosity and favouritism. As participants in the Ideology 2.0 dataset rate a different set of groups than participants in the ANES datasets, we complement the Ideology 2.0 dataset with group ideology ratings collected for this project.

### The Ideology 2.0 dataset

The Ideology 2.0 dataset (Schmidt et al., 2022) was collected from the project implicit website. Upon in‐principle acceptance of this Stage 1 registered report, we were given access to the confirmatory dataset to complete our pre‐registered analyses. Page 29 in the Supporting Information contains a detailed discussion of the Ideology 2.0 data collection procedure. The variables we use to operationalize our key constructs are detailed in Table 8.

**TABLE 8:** Summary of Ideology 2.0 measures of key constructs.

Construct	*N* items	Measure description
Animosity/Favouritism	1/Group Pair	Participants expressed a preference for one target relative to a second target in a randomly assigned target pair (e.g. liberals and conservatives, all targets given in Table SH in the Supporting Information). Measure ranged from −3 (strong preference for second target) to 3 (strong preference for first target) For Perspectives 2 and 3, the measure is recoded such that higher scores indicate preference for the conservative group in the pair, and lower scores indicate preference for the liberal group in the pair. The measure is then rescaled to range from 0 to 1. For Perspective 1, we take the absolute value of the measure to model degree of preference for one group over another regardless of group ideology. The absolute value measure is then rescaled to range from 0 to 1
Cognitive Reflection	3	Cognitive reflection was measured using the three‐item cognitive reflection test (Bialek & Pennycook, 2018; Frederick, 2005; Pennycook et al., 2015). The measure captures reflective thinking by asking participants questions that have easily accessible but incorrect answers. Participants were randomly assigned to complete between 0 and 3 items of this measure. We include participants who were assigned at least one cognitive reflection item. We imputed missing CRT responses for those who completed at least one item on the measure. Each item on the CRT is scored such that the variable takes the value of 1 if the respondent answers the question correctly, and 0 if they answer incorrectly. Composite scores on the measure were calculated by taking respondents' mean responses across the three CRT items following imputation of missing responses. We rescaled the measure to range from 0 to 1 and mean centred it
Political Ideology	1	Political ideology was measured by asking all participants, ‘What is your political identity?’ (1—Strongly conservative to 7—Strongly liberal). We reverse the measure so that higher scores indicate more conservative ideology. We then rescale the measure to range from 0 to 1 and midpoint centred it (i.e. at moderate)

*Note*: All multi‐item scales can be found on Page 31 in the Supporting Information.

We include participants (*N* = 3498) who had US citizenship, resided in the United States at the time of data collection, completed at least one item on the CRT and completed at least one relative explicit measure of group attitudes (see Table 9 for demographic characteristics). The Ideology 2.0 data collection used a planned missingness design, thus data are missing completely at random by design. We estimate the missing data using multiple imputations (see Enders, 2017). Simulation studies show that multiple imputation methods are preferred when datasets have very high levels of missingness, data are missing completely at random and if missingness on auxiliary variables is not too high. The Ideology 2.0 dataset is consistent with these requirements (e.g. Madley‐Dowd et al., 2019).

**TABLE 9:** Ideology 2.0 Demographics.

Demographics	*N*
Gender = Female	2351
Gender = Male	1147
Race = American Indian or Alaskan Native	26
Race = Black or African American	258
Race = East Asian	72
Race = More than one race – Black/White	49
Race = More than one race – Other	202
Race = Native Hawaiian or Pacific Islander	17
Race = Other or Unknown	116
Race = South Asian	49
Race = White	2709
Ethnicity = Hispanic or Latino	277
Education = Not a high school graduate	128
Education = High school graduate	202
Education = Some college or Associate's degree	1620
Education = Bachelor's degree	928
Education = Graduate degree or graduate education	620

*Note*: This table displays demographic information from the Ideology 2.0 dataset. Information is shown for the subset of the sample containing our relevant measures. This table differs slightly compared to the analogous table in the Stage 1 manuscript (Table 7). This is because a typo in our Stage 1 filtering code omitted observations relevant to two of our pre‐registered ideological groups. Upon Stage 2 confirmatory analysis, this error was recognized and fixed. Including observations relevant to these two groups increased our sample size. We also ran into an unanticipated challenge with some respondents (23 individuals) rating the same ideological groups more than once, creating concerns about practice effects and posing challenges in modelling. We excluded these 23 individuals from our final sample (this was not pre‐registered).

### Group ideology rating data collection

To estimate perceptions of each group's ideology, we used Prolific to recruit a sample of 100 people. Participants were paid $2 for their participation. We selected Prolific participants who had an approval rating greater than 95, limited the sample to US Americans, residing in the United States at the time of data collection, and aimed to recruit approximately equal numbers of men and women. Each person rated the perceived political ideology of all 21 relevant target groups on a scale ranging from 0—extremely liberal to 100—extremely conservative. These groups corresponded to the 21 politically relevant groups we analysed from the Ideology 2.0 dataset. Groups were rated and presented in a random order. We recruited 100 participants because 100 ratings are needed for reliable estimates of group ideology (e.g. Brandt & Crawford, 2016). Group ideology ratings are highly reliable across raters (see intraclass correlations [ICCs] in Studies 1 and 2; Brandt, 2017; Brandt & Crawford, 2016; ICC = 0.99). The survey also contained items relevant to other projects being conducted by our research team to conserve resources. The survey is provided on our OSF page. Informed consent was obtained from all subjects prior to data collection.

### Analyses

A description of our pre‐registered models and the observed pattern of results that would correspond to each of our three perspectives is shown in Table 10. When fitting our pre‐registered models, we ran into issues with singular fit due to lack of variation in the effect of ideological difference between groups by participant, and in intercepts by participant. Since singular fit can cause unstable estimates and inflated standard errors, we removed the participant random effects and refit our models (e.g. Bates et al., 2015). Importantly, our results are consistent across our pre‐registered and modified models, with fixed‐effect coefficients nearly identical across the specifications. As such, we report our pre‐registered models in text and report our modified models in Tables SL‐SN in the Supporting Information for interested readers.

**TABLE 10:** Hypotheses, models and predicted pattern of results for perspectives 1–3 in Study 3.

Hypothesis	Model	Terms of interest/predicted pattern of results
H1a: Individuals higher in cognitive reflection express less animosity/favouritism (without accounting for respondent ideology)	Main effects model. DV: Absolute value of preference ratings for target pairs (3 = max preference, 0 = no preference; recoded to range 0–1). IVs: Difference in group ideologies, cognitive reflection and demographic control variables	Cognitive reflection: Negative relationship suggesting that higher cognitive reflection generally corresponds to less preference for some groups over others
H1b: After accounting for respondent ideology, the effect of cognitive reflection on animosity/favouritism should reduce in magnitude	Main effects model. DV: Absolute value of preference ratings for target pairs (3 = max preference, 0 = no preference). IVs: Difference in group ideologies, cognitive reflection, *respondent ideology* and demographic control variables	Cognitive reflection: Smaller negative relationship compared to model for H1a Respondent ideology: The variable is recoded such that higher values represent more conservative ideology. Thus, there should be a positive relationship, with more conservative individuals expressing more preference for one group over another compared to liberals
H2a: Among conservatives, higher levels of cognitive reflection should predict less animosity/favouritism (i.e. the effect of target group ideology on attitudes towards ideological groups should be weaker for high cognitive reflection conservatives than low cognitive reflection conservatives)	Model with a three‐way interaction. DV: Preference ratings rescored to range from 0 to 1 such that high scores represent more preference for the conservative group and low scores represent more preference for the liberal group. 0.5 = No preference. IVs: group ideological difference, respondent ideology, cognitive reflection and demographic control variables. Two‐way interactions between group ideological difference and cognitive reflection, group ideological difference and respondent ideology, and respondent ideology and cognitive reflection. Three‐way interaction among respondent ideology, group ideological difference and cognitive reflection Simple slopes analysis for the three‐way interaction term. Examines the effect of group ideological differences on political preferences among high and low cognitive reflection liberals and conservatives	Three‐way interaction: Negative relationship indicating reflection is more predictive of preferences among liberals than conservatives Simple slopes analysis: the absolute value of the coefficient for group ideology on attitudes towards ideological groups should be *smaller* for high cognitive reflection conservatives than for lower cognitive reflection conservatives. This pattern signals they express *less* animosity/favouritism than lower cognitive reflection conservatives do
H2b: Among liberals, higher cognitive reflection should predict more animosity/favouritism (i.e. the effect of target group ideology on attitudes towards ideological groups should be stronger for high cognitive reflection liberals than low cognitive reflection liberals.)	Same model as H2a Simple slopes analysis for the three‐way interaction term. Same as above	Three‐way interaction: Negative relationship indicating reflection is more predictive of preferences among liberals than conservatives Simple slopes analysis: Absolute value of the coefficient for group ideology on attitudes towards ideological groups should be *larger* among high cognitive reflection liberals than among lower cognitive reflection liberals. This pattern signals they express *more* animosity/favouritism than low cognitive reflection liberals do
H3: Among both liberals and conservatives, high cognitive reflection should predict greater animosity/favouritism	Same model as H2 Simple slopes analysis for the three‐way interaction term. Examines the effect of target group ideology on animosity/favouritism across levels of respondent ideology and cognitive reflection	Simple slopes analysis: Absolute value of the coefficients for group ideology should be *larger* among high cognitive reflection liberals *and* conservatives than among low cognitive reflection liberals *and* conservatives. This signals that high cognitive reflection ideologues express more political group‐based preferences than lower cognitive reflection ideologues do

*Note*: All models contain a random intercept for target pair.

### Imputation with MICE

Missing data were imputed with the MICE package in R prior to analyses (van Buuren & Groothuis‐Oudshoorn, 2011). We followed current best practices and conducted 10 imputations with 10 iterations each (Stuart et al., 2009). Demographic predictors and responses to completed items were used in the imputation process. In line with our Stage 1 registered report, we used race/ethnicity, gender and education as auxiliary demographic variables. We used predictive mean matching (pmm) (Little & Rubin, 1989) to impute all continuous variables, following our Stage 1 registered report. However, due to the binary nature of the CRT responses, we deviated from our pre‐registered analysis and imputed them using logistic regression to account for their distribution. We report results with pmm imputed CRT items in Tables SI‐SK in the Supporting Information. Results are identical to those obtained using logistic regression for the binary CRT items. In conducting our imputations, four animosity/favouritism terms were collinear and thus could cause issues with unstable estimates in imputation. These terms, labour/management, foreign/local, non‐profits/corporations and socialism/capitalism, were thus all set to zero in the predictor matrix prior to imputation (van Buuren, 2018). We include more detail on this process in the replication code posted on our OSF page. All R‐hat values ranged from approximately 1 to 1.1 indicating acceptable convergence of the imputation process.

### Power analysis

We conducted MCMC power analysis using simr (Green & MacLeod, 2016) based on the results of Study 2. We possess 100% power to detect a three‐way interaction of *b* = .44 among cognitive reflection, participant ideology and target group ideological difference.

## RESULTS: STUDY 3

In contrast to Perspective 1, we find no evidence that those who are high in cognitive *reflection* express less preference for some groups over others. The coefficient for cognitive reflection is statistically non‐significant and very near zero. Adding respondent ideology to the model, the coefficient for cognitive reflection stays non‐significant and near zero. The coefficient for political ideology is also non‐significant and near zero. These results are shown in Table 11.

**TABLE 11:** Fixed effects from main effects models in the Ideology 2.0 dataset excluding and including respondent ideology.

Variable	Ideology 2.0			
	Without respondent ideology		With respondent ideology	
	*b* (SE)	β (SE)	*b* (SE)	β (SE)
Age	.03 (.016)	.01 (.007)	.03 (.015)	.01 (.007)
Ideological Difference	.32* (.14)	.25* (.11)	.32* (.14)	.25* (.11)
Education	.01 (.01)	.007 (.007)	.01 (.01)	.007 (.007)
Gender (1 = Male)	.002 (.005)	.006 (.01)	.003 (.006)	.007 (.01)
Design (1 = B)	.004 (.005)	.01 (.01)	.004 (.01)	.01 (.01)
White People vs. Non‐White People	.016* (.007)	.04* (.02)	.016* (.007)	.04* (.02)
Black People vs. Other Non‐White People	.001 (.01)	.005 (.03)	.001 (.01)	.004 (.03)
Hispanic People vs. People Except for Blacks and Whites	−.02 (.01)	−.06 (.04)	−.02* (.01)	−.06 (.04)
*Cognitive Reflection*	−.002 (.01)	−.0006 (.01)	−.0009 (.01)	−.007 (.01)
*Ideology of Respondent*	–	–	−.006 (.01)	−.005 (.01)

*Note*: The dependent variable is the absolute value of the untransformed preference variable (−3 preference for liberal group and 3 preference for conservative group) recoded to range from 0 to 1. Coefficients present in the table are rounded, whereas significance is calculated based on exact results. *p < .05. For continuous variables, standardized β coefficients represent the expected change in standard deviation units in the dependent variable per 1 *SD* unit change in the respective independent variable. Categorical variables are left untransformed.

We next fit a model with two‐way interactions between ideological differences between groups and cognitive reflection, ideological differences between groups and respondent ideology, and cognitive reflection and respondent ideology. Although our focal hypotheses are not tested by this model, we fit the model in the spirit of model building (e.g. Gelman & Hill, 2006). Because whether the liberal or conservative group in a pair is preferred is relevant in testing Perspectives 2 and 3, the dependent variable in this model and the next model we discuss range from 0 (maximum preference for the liberal group) to 1 (maximum preference for the conservative group), with a midpoint of .5 (no preference). More detail on measurement is provided in Table 10.

Replicating the worldview conflict hypothesis (e.g. Brandt et al., 2014; Brandt & Crawford, 2020), we find evidence that the effect of political ideology on preferring ideologically concordant groups is stronger when the ideological difference between groups is higher. In contrast, we find no evidence that those higher in cognitive *reflection* are more likely to prefer liberal groups over conservative groups than those lower in cognitive reflection. The interaction between cognitive reflection and respondent ideology is also non‐significant and near zero. These results are shown in Table 12.^2^

**TABLE 12:** Fixed effects of two‐way and three‐way interaction models ideology 2.0.

Variable	Two‐way interaction		Three‐way interaction	
	*b* (SE)	β (SE)	*b* (SE)	β (SE)
Age	−.01 (.02)	−.007 (.009)	−.01 (.02)	−.007 (.009)
Ideological Difference	.01 (.17)	−.11 (.16)	.01 (.17)	−.11 (.15)
Education	.0005 (.01)	.0005 (.009)	.0007 (.01)	.004 (.009)
Gender (1 = Male)	−.002 (.004)	−.005 (.01)	−.001 (.003)	−.005 (.01)
Design (1 = B)	.001 (.003)	.004 (.01)	.001 (.003)	.004 (.01)
White People vs. Non‐White People	.008 (.005)	.02 (.014)	.008 (.005)	.02 (.01)
Black People vs. Other Non‐White People Except Black People	.004 (.008)	.012 (.03)	.004 (.008)	.01 (.03)
Hispanic People vs. People Except for Blacks and Whites	−.01 (.01)	−.04 (.04)	−.01 (.01)	−.04 (.04)
Cognitive Reflection	−.007 (.01)	−.007 (.01)	−.007 (.01)	−.007 (.01)
Ideology of Respondent	.32*** (.01)	.28*** (.009)	.32*** (.01)	.28*** (.01)
Cognitive Reflection*Ideology of Respondent	.003 (.03)	.0008 (.007)	.004 (.03)	.001 (.007)
Cognitive Reflection*Ideological Difference	−.04 (.025)	−.01 (.008)	−.04 (.03)	−.01 (.008)
Ideological Difference*Ideology of Respondent	.97*** (.03)	.25*** (.01)	.97*** (.03)	.25*** (.01)
Ideological Difference* Cognitive Reflection*Ideology of Participant	–	–	.02 (.07)	.002 (.005)

*Note*: The dependent variable is preference for liberal (0) or conservative (1) group in the pair. Note that coefficients in the table are rounded, whereas significance is reported based on exact results. *p < .05, **p < .01; ***p < .001. For continuous variables, standardized β coefficients represent the expected change in standard deviation units in the dependent variable per 1 *SD* unit change in the respective independent variable. Categorical variables are left untransformed.

**FIGURE 2:**
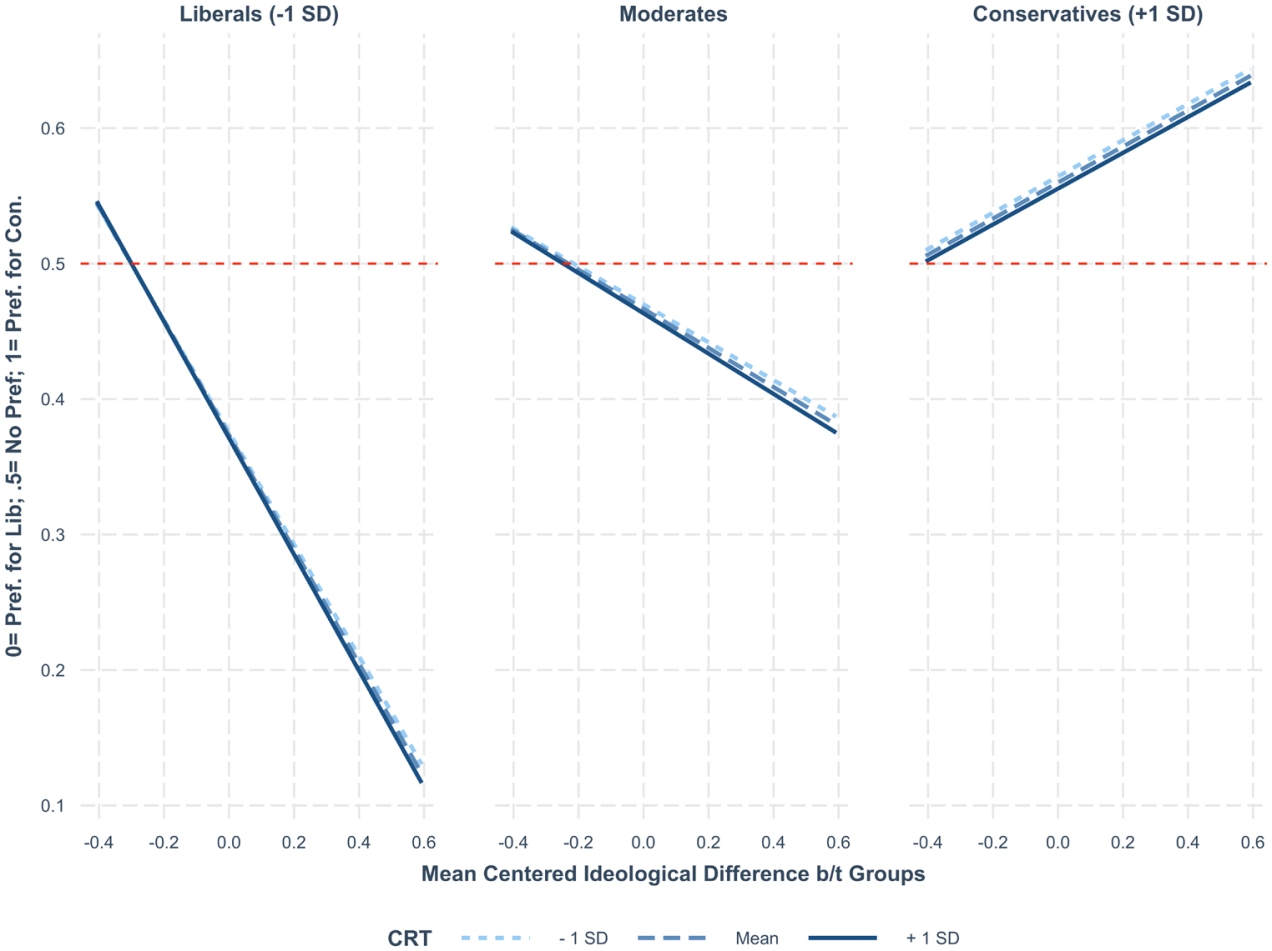
Effect of group ideological difference on relative political animosity/favouritism (group preferences) across high and low cognitive reflection liberals, conservatives and moderates. Figure 1 displays the pattern of interaction uncovered in the Ideology 2.0 dataset. Cognitive reflection does not moderate the association between group ideological differences and relative political animosity/favouritism (relative preference ratings). The figure presented here was created using one of the imputed datasets.

Finally, adding our key three‐way interaction between group ideological difference, respondent ideology and cognitive reflection for testing Perspectives 2 and 3 reveals that the three‐way interaction is near zero and non‐significant. A simple slopes analysis reveals that the relationship between target ideological difference and respondent ideology in predicting political animosity and favouritism is not significantly stronger or weaker across levels of respondent cognitive reflection. Critically, the absolute value of the coefficients for group ideological difference is not significantly smaller or larger among liberals and conservatives higher or lower in cognitive reflection. Regression results are shown in Table 12, and results of the simple slopes analysis are shown in Table 13. Figure 2 depicts the non‐significant three‐way interaction visually. These findings for cognitive *reflection* contrast with our findings for cognitive *ability*.

**TABLE 13:** Simple slopes of group ideological difference on animosity/favouritism (group preferences) for ideology 2.0 Dataset.

	Ideology 2.0					
	Liberal participants (midpoint −1 *SD*)			Conservative participants (midpoint +1 *SD*)		
	*b*	SE	β	*b*	SE	β
Lower Cognitive Reflection (Mean − 1 *SD*)	−.38*	.17	−.35*	.17	.17	.15
Higher Cognitive Reflection (Mean + 1 *SD*)	−.41*	.17	−.38*	.14	.17	.13

*Note*: Simple slopes analysis for the three‐way interaction of interest for testing Hypotheses 2–3. There is no evidence in support of either hypothesis 2 or hypothesis 3. *p < .05. Standardized β coefficients are in standard deviation units.

## DISCUSSION STUDY 3

Consistent with the results of Studies 1 and 2, we find no evidence that those lower in cognitive reflection express more political animosity than those higher in cognitive reflection. Likewise, we again find that conservatives express no more political animosity than liberals. These results are inconsistent with Perspective 1, which suggests that those lower in cognitive reflection should be *more* likely to express political animosity than those higher in cognitive reflection. The finding that conservatives express no more political animosity than liberals is also inconsistent with the predictions of Perspective 1.

We also do not find evidence consistent with Perspective 2. Perspective 2 suggests that high cognitive reflection liberals should express *more* political animosity and favouritism than low cognitive reflection liberals, while high cognitive reflection conservatives should express *less* political animosity and favouritism than low cognitive reflection conservatives.

Finally, we also find no evidence consistent with Perspective 3 in Study 3, which suggests that *high* cognitive reflection liberals and conservatives should express *more* political animosity and favouritism than low cognitive reflection liberals and conservatives. In fact, in Study 3, across all our analyses cognitive reflection was not a significant predictor of political animosity.

Importantly, the nature of our dependent variable differed between Studies 1–2 and Study 3. In Studies 1 and 2, absolute political animosity was examined (i.e. respondents rated their feelings towards one group at a time). In Study 3, relative political animosity was examined (i.e. liberals and conservatives rated their preferences for liberal and conservative target groups *relative to each other*). This raises the possibility that the difference in results between studies might stem from differences in the dependent variable, rather than genuine differences in the relationship between cognitive ability, cognitive reflection and political animosity.

The Ideology 2.0 team randomly assigned some respondents to complete absolute measures of attitudes towards ideological groups, more like those we used in Studies 1 and 2. As such, we also conducted exploratory analyses to address the possibility that differences between dependent variables explain the differences in results across studies. We fit models analogous to those we fit for Studies 1 and 2 using the Ideology 2.0 data, which contains the CRT. Our results exactly replicate the pattern of results reported in Study 3. This suggests that cognitive ability is related to greater expression of political animosity (particularly among liberals) while cognitive reflection is not. A description of the relative measures from the Ideology 2.0 dataset, the code to replicate our exploratory analyses and tables of our results are provided in the Supporting Information on our OSF page. Results pertaining to these exploratory absolute political animosity models can be found in Tables SQ‐SR.

## GENERAL DISCUSSION

Past work has examined the relationship between cognitive characteristics and attitudes among conservatives (e.g. Onraet et al., 2015), while the relationship between cognitive characteristics and attitudes among liberals is relatively unexplored. Here, we have explored whether cognitive ability and cognitive reflection contribute to the expression of political animosity in similar ways among liberals and conservatives. Across three studies (total *N* = 12,533 US Americans) we examined the relationship among cognitive ability, cognitive reflection and political animosity across ideological lines. We tested three perspectives regarding the relationship between cognitive ability, cognitive reflection and political animosity among liberals and conservatives.

The first perspective we test predicts that those lower in cognitive ability and reflection should express more political animosity than those higher in these constructs, but that the influence of cognitive ability and cognitive reflection on animosity should decline once respondent ideology is controlled for (e.g. Crandall & Eshleman, 2003; Hodson & Busseri, 2012; Jost et al., 2003). We find no support for these predictions in any of our three studies.

The second perspective we test predicts that cognitive ability and cognitive reflection should differentially predict political animosity among liberals and conservatives, displaying a particular relationship pattern with animosity across ideological lines. Specifically, among liberals, higher cognitive ability and reflection should predict expressing *more* animosity/favouritism since liberal ideology and high cognitive ability/reflection predict expressing favouritism and animosity towards the same set of groups (e.g. Brandt et al., 2014; Brandt & Crawford, 2016). Among conservatives, higher cognitive ability and reflection should predict *less* animosity, since conservatives and those high in cognitive ability/reflection express animosity and favouritism towards a different set of groups (e.g. Brandt et al., 2014; Brandt & Crawford, 2016). As such, while liberalism and high cognitive ability/reflection could reinforce each other's influence, conservatism and high cognitive ability/reflection could counteract each other's influence. While we do find that high cognitive ability liberals express more animosity than low cognitive ability liberals in Studies 1 and 2, we find no evidence that high cognitive ability conservatives express less animosity than low cognitive ability conservatives.

Finally, our third perspective predicts that those higher in cognitive ability and cognitive reflection should express more political animosity and favouritism than those lower in cognitive ability and cognitive reflection (e.g. Ganzach & Schul, 2021). We theorize that high cognitive ability and reflection could make it easier for people to acquire political knowledge, hold coherent ideologies and discern which groups in society align or conflict with their side of political debates (e.g. Carpini & Keeter, 1996; Converse, 1964; Kinder & Kalmoe, 2017). We find support for this perspective with respect to cognitive ability among liberals and conservatives in Study 1 and support for the perspective with respect to cognitive ability among liberals in Study 2. However, we find no support for the perspective with respect to cognitive reflection in Study 3.

It is interesting that we find more consistent evidence of a relationship between higher cognitive ability and greater political animosity among liberals than conservatives given the previous dearth of research on the relationship between cognitive characteristics and liberals' attitudes. While we hope this finding encourages future research into the factors that predict attitudes among liberals, future research should also probe exactly why our results with respect to cognitive ability are weaker among conservatives. For instance, it is possible that the ideological group being rated (e.g. liberals vs. conservatives vs. Black people vs. White people vs. the Tea Party vs. the LGBT+ community) may moderate the influence of cognitive ability on political animosity. Past research has found that individuals high in cognitive ability express less animosity towards groups where membership is perceived as not being a matter of choice (e.g. racial groups or sexual minority groups) than individuals low in cognitive ability (Brandt & Crawford, 2016). It is possible that greater cognitive ability is associated with greater political animosity towards high‐choice ideological outgroups such as liberals, but not towards low‐choice ideological outgroups such as racial or sexual minorities among conservatives. Such a pattern could explain why the finding that higher cognitive ability predicts increased animosity is stronger among liberals than among conservatives, as more low‐choice groups tend to be associated with political liberalism. Indeed, the group‐specific analyses presented in Tables SF‐SG in the Supporting Information from Studies 1 and 2 provide some preliminary support for this idea.

It will also be useful to identify the precise mechanism(s) accounting for the cognitive ability findings. While we present a potential mechanism that could underlie our results, our correlational analyses mean we are unable to directly test that mechanism. We propose that cognitive ability may be related to possessing the requisite political knowledge to hold a coherent political ideology, and to understand political debates in society (Carpini & Keeter, 1996). As such, those who are higher in cognitive ability may be more likely to perceive worldview conflict or alliances between themselves and ideological groups. Thus, greater perceptions of worldview conflict or ideological alliances among those who are high in cognitive ability may explain the association between high cognitive ability and the expression of political animosity. Future research should directly test this mechanism.

This potential mechanism could also help to explain why we observe a relationship between cognitive ability and political animosity but not cognitive reflection and political animosity. Political knowledge, such as understanding the difference between liberal and conservative ideologies, knowing how political institutions operate and associating groups with ideological positions, maybe more directly related to cognitive ability than cognitive reflection. Indeed, storing information in memory and efficiently accessing it is more closely related to verbal ability than it is to the tendency to override an intuitive but incorrect response (i.e. cognitive reflection; see Onraet et al., 2015; Toplak et al., 2014).

It is somewhat curious that we do not observe a relationship between cognitive reflection and *less* animosity given that past work has argued such a relationship should emerge (e.g. Blanchar & Sparkman, 2020). Future research should investigate exactly why we do not observe a relationship between cognitive reflection and lower animosity here. One possibility is that those higher in cognitive reflection are better at considering the perspectives of some outgroups (e.g. marginalized groups) than others (e.g. ideological outgroups). However, our exploratory group‐specific models from the Ideology 2.0 dataset do not seem to support this idea. Another possibility is that unusual sample characteristics of either the Ideology 2.0 dataset or the smaller convenience samples used in past research (e.g. Blanchar & Sparkman, 2020) contributed to results. Finally, it is possible that cognitive reflection predicts less use of *stereotypes* in evaluation (e.g. Blanchar & Sparkman, 2020), but is not predictive of the *valence* of attitudes towards groups (like we examine here). Future research should investigate the relationship between cognitive reflection, stereotypes and attitudes towards a wide variety of target groups (including ideological groups) in representative samples.

## CONCLUSION

We examined whether cognitive characteristics contribute to political animosity among liberals and conservatives in the same way. Here, we examine the influence of two cognitive characteristics that have been previously linked to intergroup animosity: cognitive ability and cognitive reflection. With respect to cognitive ability, we find that those higher in cognitive ability express more political animosity than those lower in cognitive ability. This finding is stronger among liberals than among conservatives. Surprisingly, we find no evidence of cognitive reflection predicting political animosity among liberals or conservatives. Future research should continue to explore how cognitive characteristics relate to political animosity across ideological lines.

## AUTHOR CONTRIBUTIONS

**Abigail L. Cassario:** Conceptualization; investigation; writing – original draft; methodology; visualization; writing – review and editing; software; formal analysis; project administration; data curation. **Shree Vallabha:** Writing – original draft; writing – review and editing; methodology. **Jordan L. Thompson:** Writing – review and editing; visualization. **Alejandro Carrillo:** Writing – review and editing; visualization. **Prachi Solanki:** Writing – review and editing. **Samantha A. Gnall:** Writing – review and editing. **Sada Rice:** Writing – review and editing. **Geoffrey A. Wetherell:** Writing – review and editing. **Mark J. Brandt:** Conceptualization; investigation; writing – review and editing; project administration; supervision; resources; methodology; funding acquisition.

## CONFLICT OF INTEREST

No authors have any conflicts of intereset to report.

## Supporting information


Data S1.

## Data Availability

Our accepted Stage 1 registered report, supplemental materials and all study data, materials and analysis code can be found on our anonymized OSF page: https://osf.io/t68z4/?view_only=d49d4f006864411a9592b8e76400eed7.

## Footnotes

^1^
We also fit group‐specific models in the full sample, and just among conservatives in both the 2012 and 2016 studies. High cognitive ability conservatives *do* express more political animosity towards most *explicitly* political groups (e.g. liberals and democrats) in both studies than low cognitive ability conservatives. This again suggests more support for Perspective 3 than Perspective 2. These results are presented in their entirety in the Tables SF and SG in the Supporting Information.

^2^
As with Studies 1 and 2, we also provide results without control variables in Tables SS‐SU in the Supporting Information. Again, our results are robust to the inclusion or exclusion of covariates.
