# Supplementary material for: Registered report: Cognitive ability, but not cognitive reflection, predicts expressing greater political animosity and favouritism
Source: Br J Soc Psychol. 2024 Nov 19;64(2):e12814. doi: 10.1111/bjso.12814 (PMC11927380; doi:10.1111/bjso.12814)
Supplement: Supplementary file 1 — Data S1. [file BJSO-64-0-s001.docx]

**Supplemental Materials for Registered Report: Cognitive Ability, But Not Cognitive Reflection Predicts Expressing Greater Political Animosity and Favouritism**

Note: We intend for these Supplemental Materials to be provided for interested readers on the osf page we link in our manuscript: <https://osf.io/t68z4/?view_only=d49d4f006864411a9592b8e76400eed7>

**Table of Contents: Information Included in Supplemental Materials**

| Groups rated Studies 1 and 2 | 3 |
| --- | --- |
| Results from Studies 1 and 2 without control variables | 5 |
| Correlation between cognitive ability and conservatism | 7 |
| Group specific models Studies 1 and 2 | 8 |
| Group specific models among conservatives only Studies 1 and 2 | 21 |
| Detailed description of Ideology2.0 data collection | 29 |
| Ideology2.0 target pairs | 31 |
| CRT items in Ideology2.0 data collection | 32 |
| Ideology2.0 results with PMM imputed CRT items | 33 |
| Ideology2.0 results without participant random effects | 37 |
| Ideology2.0 group specific models | 41 |
| Ideology2.0 group specific models among conservatives only | 46 |
| Ideology2.0 exploratory absolute measure analyses | 49 |
| Ideology2.0 results without control variables | 54 |
| Correlation between cognitive reflection and conservatism | 57 |
| Full study materials for new group ideology ratings for Ideology2.0 | Osf page |

**Groups Included in Feeling Thermometer Ratings 2012 and 2016**

**2012**

1. Christian Fundamentalists
2. Catholics
3. Feminists
4. Liberals
5. Middle Class People
6. Labor Unions
7. Poor People
8. The Military
9. Big Business
10. People on Welfare
11. Conservatives
12. Working Class People
13. Gay Men and Lesbians
14. Rich People
15. Muslims
16. Christians
17. Atheists
18. Mormons
19. Tea Party
20. Asian Americans
21. Hispanics
22. Blacks
23. Illegal Immigrants
24. Whites

**2016**

1. Christian Fundamentalists
2. Feminists
3. Poor People
4. Liberals
5. Labor Unions
6. Big Business
7. Conservatives
8. Gay Men and Lesbians
9. Muslims
10. Christians
11. Asian Americans
12. Hispanics
13. Blacks
14. Illegal Immigrants
15. Whites
16. Transgender People
17. Jews
18. Scientists
19. Police
20. Rich People
21. Tea Party

**Results from Studies 1 and 2 Without Control Variables**

| **Table SA**  *Fixed Effects 2012 and 2016 ANES Main Effects Models with No Controls No Ideology* |  |  |
| --- | --- | --- |
| **Variable** | **2016 ANES Model**  Coefficient  (Standard Error) | **2012 ANES Model**  Coefficient  (Standard Error) |
| Intercept | .39  (.03) | .43  (.03) |
| Cognitive Ability | -.002  (.008) | .03***  (.006) |
| **Table SB**  *Fixed Effects 2012 and 2016 ANES Main Effects Models with No Controls With Ideology* |  |  |
| **Variable** | **2016 ANES Model**  Coefficient  (Standard Error) | **2012 ANES Model**  Coefficient  (Standard Error) |
| Intercept | .38  (.03) | .43  (.03) |
| Cognitive Ability | .002  (.009) | .04***  (.006) |
| Ideology of Respondent | -.005  (.007) | .007  (.006) |

| **Table SC**  *Fixed Effects 2012 and 2016 ANES Models with No Controls Two Way Interaction* |  |  |
| --- | --- | --- |
| **Variable** | **2016 ANES Model**  Coefficient  (Standard Error) | **2012 ANES Model**  Coefficient  (Standard Error) |
| Intercept | .38  (.03) | .43  (.03) |
| Ideology of Group | .06  (.10) | -.03  (.13) |
| Cognitive Ability | -.008  (.008) | .03***  (.006) |
| Ideology of Respondent | .06***  (.01) | .04***  (.00) |
| Ideology of Group* Cognitive Ability | .25***  (.03) | .22***  (.02) |
| Cognitive Ability *Ideology of Respondent | -.009  (.03) | -.06*  (.03) |
| Ideology of Group*Ideology of Respondent | -1.42***  (.02) | -1.40***  (.02) |

| **Table SD**  *Fixed Effects 2012 and 2016 ANES Models with No Controls, 3 Way Interaction* |  |  |
| --- | --- | --- |
| **Variable** | **2016 ANES Model**  Coefficient  (Standard Error) | **2012 ANES Model**  Coefficient  (Standard Error) |
| Intercept | .38  (.03) | .43  (.03) |
| Ideology of Group | .05  (.10) | -.03  (.12) |
| Cognitive Ability | -.008  (.008) | .03***  (.006) |
| Ideology of Respondent | .05***  (.00) | .04***  (.00) |
| Ideology of Group* Cognitive Ability | .26***  (.03) | .22***  (.02) |
| Cognitive Ability *Ideology of Respondent | .03  (.03) | -.02  (.02) |
| Ideology of Group*Ideology of Respondent | -1.37*** (.02) | -1.37***  (.02) |
| Ideology of Group*Ideology of Respondent* Cognitive Ability | -.89***  (.10) | -1.67***  (.08) |

*Note* ***** p < .05 ** p <.01 *** p <.001

**Table SE: Correlation Between Cognitive Ability and Ideology in the 2012 and 2016 ANES Datasets**

|  |  |
| --- | --- |
| 2012 ANES | 2016 ANES |
| -.004 | -.07 |

**Table SF: Group Specific Models- 2012 and 2016 ANES**

**2012 Group Specific Results:**

## Table 1 : Christian Fundamentalists

| **term** | **estimate** | **std.error** | **statistic** | **p.value** |
| --- | --- | --- | --- | --- |
| (Intercept) | 0.533 | 0.004 | 151.743 | 0 |
| Ideology of Respondent | -0.412 | 0.014 | -28.837 | 0 |
| Cognitive Ability | 0.338 | 0.015 | 22.129 | 0 |
| Ideology of Respondent* Cognitive Ability | -0.826 | 0.061 | -13.439 | 0 |

## Table 2 : Catholics

| **term** | **estimate** | **std.error** | **statistic** | **p.value** |
| --- | --- | --- | --- | --- |
| (Intercept) | 0.401 | 0.003 | 118.003 | 0 |
| Ideology of Respondent | -0.174 | 0.014 | -12.576 | 0 |
| Cognitive Ability | 0.100 | 0.015 | 6.768 | 0 |
| Ideology of Respondent* Cognitive Ability | -0.325 | 0.059 | -5.485 | 0 |

## Table 3 : Feminists

| **term** | **estimate** | **std.error** | **statistic** | **p.value** |
| --- | --- | --- | --- | --- |
| (Intercept) | 0.483 | 0.003 | 151.600 | 0.00 |
| Ideology of Respondent | 0.369 | 0.013 | 28.446 | 0.00 |
| Cognitive Ability | 0.024 | 0.014 | 1.754 | 0.08 |
| Ideology of Respondent* Cognitive Ability | 0.538 | 0.056 | 9.627 | 0.00 |

## Table 4 : Liberals

| **term** | **estimate** | **std.error** | **statistic** | **p.value** |
| --- | --- | --- | --- | --- |
| (Intercept) | 0.504 | 0.003 | 172.520 | 0 |
| Ideology of Respondent | 0.663 | 0.012 | 55.928 | 0 |
| Cognitive Ability | 0.057 | 0.013 | 4.491 | 0 |
| Ideology of Respondent* Cognitive Ability | 0.643 | 0.051 | 12.625 | 0 |

## Table 5 : Middle Class People

| **term** | **estimate** | **std.error** | **statistic** | **p.value** |
| --- | --- | --- | --- | --- |
| (Intercept) | 0.235 | 0.003 | 90.172 | 0.000 |
| Ideology of Respondent | 0.019 | 0.011 | 1.823 | 0.068 |
| Cognitive Ability | 0.008 | 0.011 | 0.744 | 0.457 |
| Ideology of Respondent* Cognitive Ability | 0.022 | 0.045 | 0.486 | 0.627 |

## Table 6 : Labor Unions

| **term** | **estimate** | **std.error** | **statistic** | **p.value** |
| --- | --- | --- | --- | --- |
| (Intercept) | 0.496 | 0.004 | 141.674 | 0 |
| Ideology of Respondent | 0.421 | 0.014 | 29.591 | 0 |
| Cognitive Ability | 0.160 | 0.015 | 10.522 | 0 |
| Ideology of Respondent* Cognitive Ability | 0.589 | 0.061 | 9.630 | 0 |

## Table 7 : Poor People

| **term** | **estimate** | **std.error** | **statistic** | **p.value** |
| --- | --- | --- | --- | --- |
| (Intercept) | 0.306 | 0.003 | 103.963 | 0.000 |
| Ideology of Respondent | 0.085 | 0.012 | 7.103 | 0.000 |
| Cognitive Ability | 0.108 | 0.013 | 8.480 | 0.000 |
| Ideology of Respondent* Cognitive Ability | -0.001 | 0.051 | -0.020 | 0.984 |

## Table 8 : The Military

| **term** | **estimate** | **std.error** | **statistic** | **p.value** |
| --- | --- | --- | --- | --- |
| (Intercept) | 0.203 | 0.003 | 70.101 | 0 |
| Ideology of Respondent | -0.179 | 0.012 | -15.189 | 0 |
| WORDSUM | 0.122 | 0.013 | 9.664 | 0 |
| Ideology of Respondent*Cognitive Ability | -0.460 | 0.051 | -9.106 | 0 |

## Table 9 : Big Business

| **term** | **estimate** | **std.error** | **statistic** | **p.value** |
| --- | --- | --- | --- | --- |
| (Intercept) | 0.522 | 0.003 | 161.201 | 0 |
| Ideology of Respondent | -0.294 | 0.013 | -22.274 | 0 |
| Cognitive Ability | 0.158 | 0.014 | 11.234 | 0 |
| Ideology of Respondent* Cognitive Ability | -0.465 | 0.057 | -8.226 | 0 |

## Table 10 : People on Welfare

| **term** | **estimate** | **std.error** | **statistic** | **p.value** |
| --- | --- | --- | --- | --- |
| (Intercept) | 0.496 | 0.003 | 158.718 | 0.00 |
| Ideology of Respondent | 0.215 | 0.013 | 16.874 | 0.00 |
| Cognitive Ability | 0.089 | 0.014 | 6.581 | 0.00 |
| Ideology of Respondent* Cognitive Ability | 0.141 | 0.055 | 2.581 | 0.01 |

## Table 11 : Conservatives

| **term** | **estimate** | **std.error** | **statistic** | **p.value** |
| --- | --- | --- | --- | --- |
| (Intercept) | 0.484 | 0.003 | 172.105 | 0 |
| Ideology of Respondent | -0.591 | 0.011 | -51.641 | 0 |
| Cognitive Ability | 0.076 | 0.012 | 6.188 | 0 |
| Ideology of Respondent* Cognitive Ability | -0.594 | 0.049 | -12.059 | 0 |

## Table 12 : Working Class People

| **term** | **estimate** | **std.error** | **statistic** | **p.value** |
| --- | --- | --- | --- | --- |
| (Intercept) | 0.173 | 0.002 | 70.870 | 0.000 |
| Ideology of Respondent | -0.018 | 0.010 | -1.769 | 0.077 |
| Cognitive Ability | 0.080 | 0.011 | 7.542 | 0.000 |
| Ideology of Respondent* Cognitive Ability | -0.015 | 0.042 | -0.357 | 0.721 |

## Table 13 : Gay Men and Lesbians

| **term** | **estimate** | **std.error** | **statistic** | **p.value** |
| --- | --- | --- | --- | --- |
| (Intercept) | 0.467 | 0.004 | 127.345 | 0 |
| Ideology of Respondent | 0.418 | 0.015 | 27.998 | 0 |
| Cognitive Ability | -0.143 | 0.016 | -8.983 | 0 |
| Ideology of Respondent* Cognitive Ability | 0.279 | 0.064 | 4.361 | 0 |

## Table 14 : Rich People

| **term** | **estimate** | **std.error** | **statistic** | **p.value** |
| --- | --- | --- | --- | --- |
| (Intercept) | 0.502 | 0.003 | 156.210 | 0.000 |
| Ideology of Respondent | -0.236 | 0.013 | -18.056 | 0.000 |
| Cognitive Ability | -0.018 | 0.014 | -1.297 | 0.195 |
| Ideology of Respondent* Cognitive Ability | -0.332 | 0.056 | -5.922 | 0.000 |

## Table 15 : Muslims

| **term** | **estimate** | **std.error** | **statistic** | **p.value** |
| --- | --- | --- | --- | --- |
| (Intercept) | 0.549 | 0.003 | 166.433 | 0.000 |
| Ideology of Respondent | 0.238 | 0.013 | 17.734 | 0.000 |
| Cognitive Ability | -0.014 | 0.014 | -1.004 | 0.316 |
| Ideology of Respondent* Cognitive Ability | 0.144 | 0.058 | 2.504 | 0.012 |

## Table 16 : Christians

| **term** | **estimate** | **std.error** | **statistic** | **p.value** |
| --- | --- | --- | --- | --- |
| (Intercept) | 0.288 | 0.003 | 92.153 | 0 |
| Ideology of Respondent | -0.248 | 0.013 | -19.500 | 0 |
| Cognitive Ability | 0.140 | 0.014 | 10.303 | 0 |
| Ideology of Respondent* Cognitive Ability | -0.283 | 0.054 | -5.204 | 0 |

## Table 17 : Atheists

| **term** | **estimate** | **std.error** | **statistic** | **p.value** |
| --- | --- | --- | --- | --- |
| (Intercept) | 0.605 | 0.004 | 168.258 | 0 |
| Ideology of Respondent | 0.271 | 0.015 | 18.513 | 0 |
| Cognitive Ability | -0.220 | 0.016 | -14.004 | 0 |
| Ideology of Respondent* Cognitive Ability | 0.400 | 0.063 | 6.318 | 0 |

## Table 18 : Mormons

| **term** | **estimate** | **std.error** | **statistic** | **p.value** |
| --- | --- | --- | --- | --- |
| (Intercept) | 0.502 | 0.003 | 159.188 | 0 |
| Ideology of Respondent | -0.181 | 0.013 | -14.090 | 0 |
| Cognitive Ability | -0.085 | 0.014 | -6.150 | 0 |
| Ideology of Respondent* Cognitive Ability | -0.259 | 0.056 | -4.654 | 0 |

## Table 19 : Tea Party

| **term** | **estimate** | **std.error** | **statistic** | **p.value** |
| --- | --- | --- | --- | --- |
| (Intercept) | 0.604 | 0.003 | 178.843 | 0 |
| Ideology of Respondent | -0.570 | 0.014 | -41.410 | 0 |
| Cognitive Ability | 0.122 | 0.015 | 8.301 | 0 |
| Ideology of Respondent* Cognitive Ability | -0.821 | 0.059 | -13.919 | 0 |

## Table 20 : Asian American

| **term** | **estimate** | **std.error** | **statistic** | **p.value** |
| --- | --- | --- | --- | --- |
| (Intercept) | 0.339 | 0.003 | 117.352 | 0.000 |
| Ideology of Respondent | 0.048 | 0.012 | 4.076 | 0.000 |
| Cognitive Ability | -0.161 | 0.013 | -12.801 | 0.000 |
| Ideology of Respondent* Cognitive Ability | 0.028 | 0.051 | 0.546 | 0.585 |

## Table 21 : Hispanics

| **term** | **estimate** | **std.error** | **statistic** | **p.value** |
| --- | --- | --- | --- | --- |
| (Intercept) | 0.338 | 0.003 | 105.926 | 0.000 |
| Ideology of Respondent | 0.107 | 0.013 | 8.233 | 0.000 |
| Cognitive Ability | -0.081 | 0.014 | -5.841 | 0.000 |
| Ideology of Respondent* Cognitive Ability | 0.013 | 0.056 | 0.237 | 0.813 |

## Table 22 : Blacks

| **term** | **estimate** | **std.error** | **statistic** | **p.value** |
| --- | --- | --- | --- | --- |
| (Intercept) | 0.328 | 0.003 | 103.382 | 0.000 |
| Ideology of Respondent | 0.127 | 0.013 | 9.838 | 0.000 |
| Cognitive Ability | 0.006 | 0.014 | 0.455 | 0.649 |
| Ideology of Respondent* Cognitive Ability | 0.032 | 0.055 | 0.581 | 0.561 |

## Table 23 : Illegal Immigrants

| **term** | **estimate** | **std.error** | **statistic** | **p.value** |
| --- | --- | --- | --- | --- |
| (Intercept) | 0.587 | 0.004 | 157.711 | 0.000 |
| Ideology of Respondent | 0.311 | 0.015 | 20.562 | 0.000 |
| Cognitive Ability | 0.048 | 0.016 | 2.942 | 0.003 |
| Ideology of Respondent* Cognitive Ability | 0.275 | 0.065 | 4.215 | 0.000 |

## Table 24 : Whites

| **term** | **estimate** | **std.error** | **statistic** | **p.value** |
| --- | --- | --- | --- | --- |
| (Intercept) | 0.280 | 0.003 | 100.368 | 0.000 |
| Ideology of Respondent | -0.039 | 0.011 | -3.447 | 0.001 |
| Cognitive Ability | -0.078 | 0.012 | -6.458 | 0.000 |
| Ideology of Respondent* Cognitive Ability | 0.080 | 0.049 | 1.634 | 0.102 |

**2016 Group Specific Results:**

## Table 1 : Christian Fundamentalists

| **term** | **estimate** | **std.error** | **statistic** | **p.value** |
| --- | --- | --- | --- | --- |
| (Intercept) | 0.512 | 0.005 | 111.064 | 0 |
| Ideology of Respondent | -0.485 | 0.018 | -27.380 | 0 |
| Cognitive Ability | 0.309 | 0.021 | 14.999 | 0 |
| Ideology of Respondent* Cognitive Ability | -0.448 | 0.079 | -5.664 | 0 |

## Table 2 : Feminists

| **term** | **estimate** | **std.error** | **statistic** | **p.value** |
| --- | --- | --- | --- | --- |
| (Intercept) | 0.429 | 0.004 | 99.764 | 0 |
| Ideology of Respondent | 0.486 | 0.017 | 29.366 | 0 |
| Cognitive Ability | -0.079 | 0.019 | -4.071 | 0 |
| Ideology of Respondent* Cognitive Ability | 0.529 | 0.074 | 7.123 | 0 |

## Table 3 : Liberals

| **term** | **estimate** | **std.error** | **statistic** | **p.value** |
| --- | --- | --- | --- | --- |
| (Intercept) | 0.468 | 0.004 | 119.583 | 0.00 |
| Ideology of Respondent | 0.687 | 0.015 | 45.738 | 0.00 |
| Cognitive Ability | -0.019 | 0.018 | -1.057 | 0.29 |
| Ideology of Respondent* Cognitive Ability | 0.319 | 0.067 | 4.738 | 0.00 |

## Table 4 : Labor Unions

| **term** | **estimate** | **std.error** | **statistic** | **p.value** |
| --- | --- | --- | --- | --- |
| (Intercept) | 0.433 | 0.004 | 100.016 | 0 |
| Ideology of Respondent | 0.345 | 0.017 | 20.727 | 0 |
| Cognitive Ability | 0.121 | 0.019 | 6.249 | 0 |
| Ideology of Respondent* Cognitive Ability | 0.403 | 0.074 | 5.427 | 0 |

## Table 5 : Poor People

| **term** | **estimate** | **std.error** | **statistic** | **p.value** |
| --- | --- | --- | --- | --- |
| (Intercept) | 0.275 | 0.004 | 74.122 | 0.000 |
| Ideology of Respondent | 0.050 | 0.014 | 3.478 | 0.001 |
| Cognitive Ability | 0.002 | 0.017 | 0.135 | 0.892 |
| Ideology of Respondent* Cognitive Ability | 0.175 | 0.063 | 2.757 | 0.006 |

## Table 6 : Big Business

| **term** | **estimate** | **std.error** | **statistic** | **p.value** |
| --- | --- | --- | --- | --- |
| (Intercept) | 0.505 | 0.004 | 125.371 | 0 |
| Ideology of Respondent | -0.266 | 0.015 | -17.236 | 0 |
| Cognitive Ability | 0.117 | 0.018 | 6.474 | 0 |
| Ideology of Respondent* Cognitive Ability | -0.309 | 0.069 | -4.480 | 0 |

## Table 7 : Conservatives

| **term** | **estimate** | **std.error** | **statistic** | **p.value** |
| --- | --- | --- | --- | --- |
| (Intercept) | 0.446 | 0.004 | 118.063 | 0 |
| Ideology of Respondent | -0.621 | 0.015 | -42.703 | 0 |
| Cognitive Ability | 0.069 | 0.017 | 4.092 | 0 |
| Ideology of Respondent* Cognitive Ability | -0.326 | 0.065 | -5.010 | 0 |

## Table 8 : Gay Men and Lesbians

| **term** | **estimate** | **std.error** | **statistic** | **p.value** |
| --- | --- | --- | --- | --- |
| (Intercept) | 0.372 | 0.005 | 82.022 | 0.000 |
| Ideology of Respondent | 0.472 | 0.017 | 27.022 | 0.000 |
| Cognitive Ability | -0.165 | 0.020 | -8.145 | 0.000 |
| Ideology of Respondent* Cognitive Ability | 0.074 | 0.078 | 0.941 | 0.347 |

## Table 9 : Muslims

| **term** | **estimate** | **std.error** | **statistic** | **p.value** |
| --- | --- | --- | --- | --- |
| (Intercept) | 0.438 | 0.004 | 97.322 | 0.00 |
| Ideology of Respondent | 0.363 | 0.017 | 20.978 | 0.00 |
| Cognitive Ability | -0.088 | 0.020 | -4.357 | 0.00 |
| Ideology of Respondent* Cognitive Ability | 0.179 | 0.077 | 2.324 | 0.02 |

## Table 10 : Christians

| **term** | **estimate** | **std.error** | **statistic** | **p.value** |
| --- | --- | --- | --- | --- |
| (Intercept) | 0.255 | 0.004 | 60.329 | 0.000 |
| Ideology of Respondent | -0.331 | 0.016 | -20.364 | 0.000 |
| Cognitive Ability | 0.073 | 0.019 | 3.881 | 0.000 |
| Ideology of Respondent* Cognitive Ability | -0.151 | 0.072 | -2.082 | 0.037 |

## Table 11 : Asian American

| **term** | **estimate** | **std.error** | **statistic** | **p.value** |
| --- | --- | --- | --- | --- |
| (Intercept) | 0.293 | 0.004 | 77.863 | 0.000 |
| Ideology of Respondent | 0.081 | 0.014 | 5.600 | 0.000 |
| Cognitive Ability | -0.134 | 0.017 | -7.897 | 0.000 |
| Ideology of Respondent* Cognitive Ability | 0.085 | 0.065 | 1.318 | 0.188 |

## Table 12 : Hispanics

| **term** | **estimate** | **std.error** | **statistic** | **p.value** |
| --- | --- | --- | --- | --- |
| (Intercept) | 0.301 | 0.004 | 76.993 | 0.000 |
| Ideology of Respondent | 0.121 | 0.015 | 8.069 | 0.000 |
| Cognitive Ability | -0.084 | 0.018 | -4.772 | 0.000 |
| Ideology of Respondent* Cognitive Ability | 0.121 | 0.067 | 1.794 | 0.073 |

## Table 13 : Blacks

| **term** | **estimate** | **std.error** | **statistic** | **p.value** |
| --- | --- | --- | --- | --- |
| (Intercept) | 0.306 | 0.004 | 77.803 | 0.000 |
| Ideology of Respondent | 0.151 | 0.015 | 10.004 | 0.000 |
| Cognitive Ability | -0.048 | 0.018 | -2.721 | 0.007 |
| Ideology of Respondent* Cognitive Ability | 0.040 | 0.068 | 0.595 | 0.552 |

## Table 14 : Illegal Immigrants

| **term** | **estimate** | **std.error** | **statistic** | **p.value** |
| --- | --- | --- | --- | --- |
| (Intercept) | 0.573 | 0.005 | 120.373 | 0.000 |
| Ideology of Respondent | 0.430 | 0.018 | 23.549 | 0.000 |
| Cognitive Ability | 0.016 | 0.021 | 0.761 | 0.447 |
| Ideology of Respondent* Cognitive Ability | 0.270 | 0.082 | 3.300 | 0.001 |

## Table 15 : Whites

| **term** | **estimate** | **std.error** | **statistic** | **p.value** |
| --- | --- | --- | --- | --- |
| (Intercept) | 0.279 | 0.004 | 75.648 | 0.000 |
| Ideology of Respondent | -0.082 | 0.014 | -5.749 | 0.000 |
| Cognitive Ability | -0.031 | 0.017 | -1.895 | 0.058 |
| Ideology of Respondent* Cognitive Ability | 0.037 | 0.064 | 0.581 | 0.561 |

## Table 16 : Transgender People

| **term** | **estimate** | **std.error** | **statistic** | **p.value** |
| --- | --- | --- | --- | --- |
| (Intercept) | 0.426 | 0.005 | 93.617 | 0.000 |
| Ideology of Respondent | 0.493 | 0.018 | 28.043 | 0.000 |
| Cognitive Ability | -0.124 | 0.020 | -6.064 | 0.000 |
| Ideology of Respondent* Cognitive Ability | 0.051 | 0.079 | 0.639 | 0.523 |

## Table 17 : Jews

| **term** | **estimate** | **std.error** | **statistic** | **p.value** |
| --- | --- | --- | --- | --- |
| (Intercept) | 0.276 | 0.004 | 69.626 | 0.000 |
| Ideology of Respondent | 0.027 | 0.015 | 1.796 | 0.073 |
| Cognitive Ability | -0.148 | 0.018 | -8.379 | 0.000 |
| Ideology of Respondent* Cognitive Ability | 0.176 | 0.068 | 2.605 | 0.009 |

## Table 18 : Scientists

| **term** | **estimate** | **std.error** | **statistic** | **p.value** |
| --- | --- | --- | --- | --- |
| (Intercept) | 0.222 | 0.004 | 62.708 | 0.000 |
| Ideology of Respondent | 0.193 | 0.014 | 14.197 | 0.000 |
| Cognitive Ability | -0.085 | 0.016 | -5.367 | 0.000 |
| Ideology of Respondent* Cognitive Ability | 0.209 | 0.061 | 3.445 | 0.001 |

## Table 19 : Police

| **term** | **estimate** | **std.error** | **statistic** | **p.value** |
| --- | --- | --- | --- | --- |
| (Intercept) | 0.249 | 0.004 | 61.832 | 0.000 |
| Ideology of Respondent | -0.270 | 0.016 | -17.397 | 0.000 |
| Cognitive Ability | -0.057 | 0.018 | -3.172 | 0.002 |
| Ideology of Respondent* Cognitive Ability | -0.012 | 0.069 | -0.168 | 0.867 |

## Table 20 : Rich

| **term** | **estimate** | **std.error** | **statistic** | **p.value** |
| --- | --- | --- | --- | --- |
| (Intercept) | 0.458 | 0.004 | 120.251 | 0.000 |
| Ideology of Respondent | -0.184 | 0.015 | -12.585 | 0.000 |
| Cognitive Ability | -0.030 | 0.017 | -1.752 | 0.080 |
| Ideology of Respondent* Cognitive Ability | -0.043 | 0.065 | -0.665 | 0.506 |

## Table 21 : Tea Party

| **term** | **estimate** | **std.error** | **statistic** | **p.value** |
| --- | --- | --- | --- | --- |
| (Intercept) | 0.567 | 0.004 | 133.385 | 0 |
| Ideology of Respondent | -0.515 | 0.016 | -31.628 | 0 |
| Cognitive Ability | 0.209 | 0.019 | 10.988 | 0 |
| Ideology of Respondent* Cognitive Ability | -0.729 | 0.073 | -10.035 | 0 |

**Table SG: Group Specific Models Among Conservatives Only Studies 1 and 2**

**2012 Results:**

## Table 1 : Christian Fundamentalists

| **term** | **estimate** | **std.error** | **statistic** | **p.value** |
| --- | --- | --- | --- | --- |
| (Intercept) | 0.413 | 0.006 | 69.215 | 0 |
| Cognitive Ability | 0.112 | 0.028 | 4.057 | 0 |

## Table 2 : Catholics

| **term** | **estimate** | **std.error** | **statistic** | **p.value** |
| --- | --- | --- | --- | --- |
| (Intercept) | 0.342 | 0.005 | 64.557 | 0.000 |
| Cognitive Ability | 0.019 | 0.025 | 0.778 | 0.437 |

## Table 3 : Feminists

| **term** | **estimate** | **std.error** | **statistic** | **p.value** |
| --- | --- | --- | --- | --- |
| (Intercept) | 0.585 | 0.006 | 105.211 | 0 |
| Cognitive Ability | 0.177 | 0.026 | 6.850 | 0 |

## Table 4 : Liberals

| **term** | **estimate** | **std.error** | **statistic** | **p.value** |
| --- | --- | --- | --- | --- |
| (Intercept) | 0.694 | 0.006 | 124.301 | 0 |
| Cognitive Ability | 0.255 | 0.026 | 9.863 | 0 |

## Table 5 : Middle Class People

| **term** | **estimate** | **std.error** | **statistic** | **p.value** |
| --- | --- | --- | --- | --- |
| (Intercept) | 0.236 | 0.004 | 56.519 | 0.000 |
| Cognitive Ability | 0.044 | 0.019 | 2.269 | 0.023 |

## Table 6 : Labor Unions

| **term** | **estimate** | **std.error** | **statistic** | **p.value** |
| --- | --- | --- | --- | --- |
| (Intercept) | 0.622 | 0.006 | 101.714 | 0 |
| Cognitive Ability | 0.357 | 0.028 | 12.618 | 0 |

## Table 7 : Poor People

| **term** | **estimate** | **std.error** | **statistic** | **p.value** |
| --- | --- | --- | --- | --- |
| (Intercept) | 0.329 | 0.005 | 67.667 | 0 |
| Cognitive Ability | 0.112 | 0.022 | 4.972 | 0 |

## Table 8 : The Military

| **term** | **estimate** | **std.error** | **statistic** | **p.value** |
| --- | --- | --- | --- | --- |
| (Intercept) | 0.153 | 0.004 | 36.488 | 0.000 |
| Cognitive Ability | 0.032 | 0.019 | 1.650 | 0.099 |

## Table 9 : Big Business

| **term** | **estimate** | **std.error** | **statistic** | **p.value** |
| --- | --- | --- | --- | --- |
| (Intercept) | 0.427 | 0.005 | 81.266 | 0.000 |
| Cognitive Ability | 0.050 | 0.024 | 2.063 | 0.039 |

## Table 10 : People on Welfare

| **term** | **estimate** | **std.error** | **statistic** | **p.value** |
| --- | --- | --- | --- | --- |
| (Intercept) | 0.550 | 0.005 | 106.793 | 0 |
| Cognitive Ability | 0.146 | 0.024 | 6.110 | 0 |

## Table 11 : Conservatives

| **term** | **estimate** | **std.error** | **statistic** | **p.value** |
| --- | --- | --- | --- | --- |
| (Intercept) | 0.303 | 0.005 | 62.667 | 0.000 |
| Cognitive Ability | -0.075 | 0.022 | -3.337 | 0.001 |

## Table 12 : Working Class People

| **term** | **estimate** | **std.error** | **statistic** | **p.value** |
| --- | --- | --- | --- | --- |
| (Intercept) | 0.166 | 0.004 | 42.894 | 0 |
| Cognitive Ability | 0.102 | 0.018 | 5.696 | 0 |

## Table 13 : Gay Men and Lesbians

| **term** | **estimate** | **std.error** | **statistic** | **p.value** |
| --- | --- | --- | --- | --- |
| (Intercept) | 0.583 | 0.006 | 94.315 | 0.000 |
| Cognitive Ability | -0.065 | 0.029 | -2.282 | 0.023 |

## Table 14 : Rich People

| **term** | **estimate** | **std.error** | **statistic** | **p.value** |
| --- | --- | --- | --- | --- |
| (Intercept) | 0.421 | 0.005 | 83.961 | 0 |
| Cognitive Ability | -0.097 | 0.023 | -4.192 | 0 |

## Table 15 : Muslims

| **term** | **estimate** | **std.error** | **statistic** | **p.value** |
| --- | --- | --- | --- | --- |
| (Intercept) | 0.614 | 0.006 | 107.269 | 0.000 |
| Cognitive Ability | 0.018 | 0.027 | 0.666 | 0.506 |

## Table 16 : Christians

| **term** | **estimate** | **std.error** | **statistic** | **p.value** |
| --- | --- | --- | --- | --- |
| (Intercept) | 0.214 | 0.005 | 44.253 | 0.000 |
| Cognitive Ability | 0.062 | 0.022 | 2.787 | 0.005 |

## Table 17 : Atheists

| **term** | **estimate** | **std.error** | **statistic** | **p.value** |
| --- | --- | --- | --- | --- |
| (Intercept) | 0.677 | 0.006 | 115.124 | 0 |
| Cognitive Ability | -0.114 | 0.027 | -4.173 | 0 |

## Table 18 : Mormons

| **term** | **estimate** | **std.error** | **statistic** | **p.value** |
| --- | --- | --- | --- | --- |
| (Intercept) | 0.444 | 0.005 | 85.255 | 0 |
| Cognitive Ability | -0.149 | 0.024 | -6.166 | 0 |

## Table 19 : Tea Party

| **term** | **estimate** | **std.error** | **statistic** | **p.value** |
| --- | --- | --- | --- | --- |
| (Intercept) | 0.435 | 0.006 | 72.233 | 0 |
| Cognitive Ability | -0.107 | 0.028 | -3.860 | 0 |

## Table 20 : Asian American

| **term** | **estimate** | **std.error** | **statistic** | **p.value** |
| --- | --- | --- | --- | --- |
| (Intercept) | 0.344 | 0.005 | 72.895 | 0 |
| Cognitive Ability | -0.143 | 0.022 | -6.498 | 0 |

## Table 21 : Hispanics

| **term** | **estimate** | **std.error** | **statistic** | **p.value** |
| --- | --- | --- | --- | --- |
| (Intercept) | 0.359 | 0.005 | 70.226 | 0.000 |
| Cognitive Ability | -0.060 | 0.024 | -2.547 | 0.011 |

## Table 22 : Blacks

| **term** | **estimate** | **std.error** | **statistic** | **p.value** |
| --- | --- | --- | --- | --- |
| (Intercept) | 0.359 | 0.005 | 69.747 | 0.000 |
| Cognitive Ability | 0.037 | 0.024 | 1.530 | 0.126 |

## Table 23 : Illegal Immigrants

| **term** | **estimate** | **std.error** | **statistic** | **p.value** |
| --- | --- | --- | --- | --- |
| (Intercept) | 0.668 | 0.006 | 111.454 | 0 |
| Cognitive Ability | 0.118 | 0.028 | 4.246 | 0 |

## Table 24 : Whites

| **term** | **estimate** | **std.error** | **statistic** | **p.value** |
| --- | --- | --- | --- | --- |
| (Intercept) | 0.263 | 0.004 | 60.344 | 0.000 |
| Cognitive Ability | -0.030 | 0.020 | -1.483 | 0.138 |

**2016 Results**

## Table 1 : Christian Fundamentalists

| **term** | **estimate** | **std.error** | **statistic** | **p.value** |
| --- | --- | --- | --- | --- |
| (Intercept) | 0.374 | 0.008 | 49.725 | 0 |
| Cognitive Ability | 0.170 | 0.036 | 4.730 | 0 |

## Table 2 : Feminists

| **term** | **estimate** | **std.error** | **statistic** | **p.value** |
| --- | --- | --- | --- | --- |
| (Intercept) | 0.567 | 0.007 | 76.795 | 0.000 |
| Cognitive Ability | 0.071 | 0.035 | 2.010 | 0.045 |

## Table 3 : Liberals

| **term** | **estimate** | **std.error** | **statistic** | **p.value** |
| --- | --- | --- | --- | --- |
| (Intercept) | 0.666 | 0.007 | 93.214 | 0.000 |
| Cognitive Ability | 0.109 | 0.034 | 3.187 | 0.001 |

## Table 4 : Labor Unions

| **term** | **estimate** | **std.error** | **statistic** | **p.value** |
| --- | --- | --- | --- | --- |
| (Intercept) | 0.536 | 0.007 | 73.947 | 0 |
| Cognitive Ability | 0.224 | 0.035 | 6.473 | 0 |

## Table 5 : Poor People

| **term** | **estimate** | **std.error** | **statistic** | **p.value** |
| --- | --- | --- | --- | --- |
| (Intercept) | 0.289 | 0.006 | 48.573 | 0.000 |
| Cognitive Ability | 0.026 | 0.028 | 0.919 | 0.358 |

## Table 6 : Big Business

| **term** | **estimate** | **std.error** | **statistic** | **p.value** |
| --- | --- | --- | --- | --- |
| (Intercept) | 0.426 | 0.006 | 69.083 | 0.00 |
| Cognitive Ability | 0.034 | 0.029 | 1.151 | 0.25 |

## Table 7 : Conservatives

| **term** | **estimate** | **std.error** | **statistic** | **p.value** |
| --- | --- | --- | --- | --- |
| (Intercept) | 0.264 | 0.006 | 43.447 | 0.000 |
| Cognitive Ability | -0.041 | 0.029 | -1.410 | 0.159 |

## Table 8 : Gay Men and Lesbians

| **term** | **estimate** | **std.error** | **statistic** | **p.value** |
| --- | --- | --- | --- | --- |
| (Intercept) | 0.495 | 0.008 | 63.584 | 0 |
| Cognitive Ability | -0.149 | 0.037 | -3.998 | 0 |

## Table 9 : Muslims

| **term** | **estimate** | **std.error** | **statistic** | **p.value** |
| --- | --- | --- | --- | --- |
| (Intercept) | 0.531 | 0.008 | 70.588 | 0.000 |
| Cognitive Ability | -0.007 | 0.036 | -0.207 | 0.836 |

## Table 10 : Christians

| **term** | **estimate** | **std.error** | **statistic** | **p.value** |
| --- | --- | --- | --- | --- |
| (Intercept) | 0.159 | 0.006 | 27.574 | 0.000 |
| Cognitive Ability | 0.029 | 0.027 | 1.071 | 0.284 |

## Table 11 : Asian American

| **term** | **estimate** | **std.error** | **statistic** | **p.value** |
| --- | --- | --- | --- | --- |
| (Intercept) | 0.305 | 0.006 | 53.080 | 0 |
| Cognitive Ability | -0.109 | 0.028 | -3.953 | 0 |

## Table 12 : Hispanics

| **term** | **estimate** | **std.error** | **statistic** | **p.value** |
| --- | --- | --- | --- | --- |
| (Intercept) | 0.328 | 0.006 | 52.849 | 0.000 |
| Cognitive Ability | -0.045 | 0.030 | -1.506 | 0.132 |

## Table 13 : Blacks

| **term** | **estimate** | **std.error** | **statistic** | **p.value** |
| --- | --- | --- | --- | --- |
| (Intercept) | 0.343 | 0.006 | 55.355 | 0.000 |
| Cognitive Ability | -0.045 | 0.030 | -1.500 | 0.134 |

## Table 14 : Illegal Immigrants

| **term** | **estimate** | **std.error** | **statistic** | **p.value** |
| --- | --- | --- | --- | --- |
| (Intercept) | 0.691 | 0.008 | 91.311 | 0.000 |
| Cognitive Ability | 0.096 | 0.036 | 2.656 | 0.008 |

## Table 15 : Whites

| **term** | **estimate** | **std.error** | **statistic** | **p.value** |
| --- | --- | --- | --- | --- |
| (Intercept) | 0.251 | 0.005 | 46.320 | 0.000 |
| Cognitive Ability | -0.026 | 0.026 | -1.001 | 0.317 |

## Table 16 : Transgender People

| **term** | **estimate** | **std.error** | **statistic** | **p.value** |
| --- | --- | --- | --- | --- |
| (Intercept) | 0.553 | 0.008 | 72.089 | 0.000 |
| Cognitive Ability | -0.093 | 0.037 | -2.529 | 0.012 |

## Table 17 : Jews

| **term** | **estimate** | **std.error** | **statistic** | **p.value** |
| --- | --- | --- | --- | --- |
| (Intercept) | 0.269 | 0.006 | 43.965 | 0.000 |
| Cognitive Ability | -0.092 | 0.029 | -3.145 | 0.002 |

## Table 18 : Scientists

| **term** | **estimate** | **std.error** | **statistic** | **p.value** |
| --- | --- | --- | --- | --- |
| (Intercept) | 0.272 | 0.006 | 45.188 | 0.000 |
| Cognitive Ability | -0.011 | 0.029 | -0.367 | 0.714 |

## Table 19 : Police

| **term** | **estimate** | **std.error** | **statistic** | **p.value** |
| --- | --- | --- | --- | --- |
| (Intercept) | 0.170 | 0.006 | 30.60 | 0.000 |
| Cognitive Ability | -0.026 | 0.027 | -0.98 | 0.327 |

## Table 20 : Rich

| **term** | **estimate** | **std.error** | **statistic** | **p.value** |
| --- | --- | --- | --- | --- |
| (Intercept) | 0.396 | 0.006 | 68.913 | 0.000 |
| Cognitive Ability | -0.014 | 0.027 | -0.503 | 0.615 |

## Table 21 : Tea Party

| **term** | **estimate** | **std.error** | **statistic** | **p.value** |
| --- | --- | --- | --- | --- |
| (Intercept) | 0.416 | 0.007 | 60.570 | 0.000 |
| Cognitive Ability | -0.014 | 0.033 | -0.429 | 0.668 |

**Supplemental Materials: Detailed Discussion of Ideology2.0 Data Collection Procedure**

The Ideology2.0 dataset (Schmidt et al., 2022) was collected from the Project Implicit website. In late 2022, the proprietors of the dataset released a call for registered reports. Along with the call they released 22 percent of the dataset to facilitate the writing of pseudo code, and a masked version of the full dataset to facilitate sample size analysis. They withheld from authors, however, a confirmatory dataset which will only be provided if our Stage 1registered report receives in principle acceptance. It is this confirmatory dataset, to which we have not had prior access, that we will use in Study 3. A letter from the proprietors of the Ideology2.0 dataset attesting that we have not had prior access to the confirmatory data was provided to the BJPS editor in chief prior to our submission of this registered report. Below, we provide a detailed description of the Ideology2.0 study design.

Participants were people who visited the Project Implicit website and were randomly assigned to complete the Ideolgoy2.0 study, which was one of several studies fielded by Project Implicit researchers. First, participants provided basic demographic information and session information was recorded. Participants were then randomly assigned to one of two study designs. Participants assigned to design A completed implicit and explicit measures of 1 of 52 possible topics. Implicit and explicit measures included a standard or single target IAT, a 1-item relative preference (or liking) measure, and 8 of 20 possible explicit measures. They were then randomly assigned to complete 4 items each from 5 of 25 possible scales or sets of scales, and 8 items from a pool of 186 items. Participants assigned to design B completed implicit and explicit measures for 2 of 52 possible topics including standard and single-topic IATs and a 1-item relative preference (or liking) measure. They were then randomly assigned to complete 4 items each from 2 of 25 possible scales, and 6 items each from a pool of 186 items. Although participants assigned to design B have multiple rows in the dataset, they have anonymized user IDs that can be used to track and account for repeated observations. We detail how we account for these repeated observations when discussing our modeling strategy in the manuscript. In short, we handle this by including a random intercept for participant in our multilevel models.

**Target Pairs in Ideology2.0 Dataset**

| **Table SH**  *Target Groups in Ideology2.0 Data* |  |
| --- | --- |
| Target 1 | Target 2 |
| Gay people | Straight people |
| Non-Profits | Corporations |
| Labor | Management |
| Foreign people | Local people |
| Black people | White people |
| Mothers | Fathers |
| Democrats | Republicans |
| Liberals | Conservatives |
| Religious People | Atheists |
| Religion | Science |
| Capitalism | Socialism |

**Supplemental Materials: CRT Items**

1. A bat and a ball cost $1.10 in total. The bat costs $1 more than the ball. How much does the ball cost?
2. If it takes 5 identical machines 5 minutes to make 5 widgets, how long would it take 1 machine to make 1 widget?
3. In a lake, there is a patch of lily pads. Every day, the patch doubles in size. If it takes 48 days for the patch to cover the entire lake, how long would it take for it to cover half the lake?

**Table SI**

*Fixed Effects from Main Effects Models in the Ideology2.0 Dataset Excluding and Including Respondent Ideology for PMM Imputed CRT*

|  | **Ideology2.0** | | | |
| --- | --- | --- | --- | --- |
|  | **Without Respondent Ideology** | | **With Respondent Ideology** | |
| **Variable** | b  (SE) | β  (SE) | b  (SE) | β  (SE) |
| Age | .03  (.017) | .01  (.007) | .03  (.016) | .01  (.008) |
| Ideological Difference | .34*  (.15) | .27*  (.12) | .34*  (.14) | .27*  (.12) |
| Education | .01  (.01) | .007  (.007) | .01  (.01) | .008  (.007) |
| Gender (1= Male) | .0004  (.005) | .007  (.01) | .0007  (.006) | .002  (.01) |
| Design (1= B) | .003  (.004) | .007  (.01) | .003  (.004) | .006  (.01) |
| White People v. Non-White People | .014  (.008) | .04  (.02) | .014  (.007) | .04  (.02) |
| Black People vs. Other Non-White People Except Whites | -.005  (.01) | -.001  (.03) | -.005  (.01) | -.001  (.03) |
| Hispanic People vs. Other Non-White People Except Blacks and Whites | -.03  (.01) | -.07  (.04) | -.02  (.01) | -.07  (.04) |
| **Cognitive Reflection** | .001  (.01) | .0009  (.01) | .0009  (.01) | .0008  (.01) |
| **Ideology of Respondent** | - | - | -.004  (.01) | -.003  (.01) |

*Note:* The dependent variable is the absolute value of the untransformed preference variable (-3 preference for liberal group, 3 preference for conservative group) recoded to range from 0-1. *p< .05, **p < .01; ***p < .001. *b* represents coefficients for models where variables are rescaled to range from 0-1 β represents coefficients for models where variables are standardized by standard deviation units (z-scored). For continuous variables, β coefficients represent the expected change in standard deviation units in the dependent variable per one standard deviation unit change in the respective independent variable. For categorical variables, β represents expected standard deviation change in the dependent variable if a member of the category.

**Table SJ**

*Fixed Effects of Two-Way and Three-Way Interaction Models Ideology2.0 PMM Imputed Data*

|  | **Two Way Interaction** | | **Three Way Interaction** | |
| --- | --- | --- | --- | --- |
| **Variable** | b  (SE) | β  (SE) | b  (SE) | β  (SE) |
| Age | -.01  (.01) | -.007  (.007) | -.01  (.01) | -.007  (.007) |
| Ideological Difference | .001  (.17) | -.12  (.16) | .002  (.17) | -.12  (.16) |
| Education | .0003  (.01) | .0001  (.009) | .0003  (.01) | .0001  (.009) |
| Gender (1= Male) | -.002  (.004) | -.0004  (.01) | -.002  (.004) | -.00005  (.01) |
| Design (1= B) | .001 (.003) | .004  (.01) | .001  (.004) | .004  (.01) |
| White People v. Non-White People | .006  (.006) | .02  (.02) | .006  (.007) | .02  (.02) |
| Black People vs. Other Non-White People Except Whites | .007  (.009) | .02  (.03) | .008  (.009) | .02  (.03) |
| Hispanic People vs. Other Non-White People Except Blacks and Whites | -.01  (.01) | -.04*  (.04) | -.01  (.01) | -.04  (.03) |
| Cognitive Reflection | -.01  (.01) | -.01  (.01) | -.01  (.01) | -.01  (.01) |
| Ideology of Respondent | .31***  (.02) | .28***  (.02) | .32***  (.02) | .28***  (.02) |
| Cognitive Reflection*Ideology of Respondent | .01  (.03) | .004  (.007) | .01  (.03) | .005  (.008) |
| Cognitive Reflection*Ideological Difference | -.05  (.04) | -.01  (.01) | -.04  (.04) | -.01  (.01) |
| Ideological Difference*Ideology of Respondent | .97***  (.04) | .26***  (.01) | .97***  (.04) | .26***  (.01) |
| **Ideological Difference* Cognitive Reflection*Ideology of Participant** | **-** | **-** | **.03**  **(.07)** | **.002**  **(.006)** |

*Note:* The outcome variable is preference for liberal (0) or conservative (1) group in the pair. *p < .05, **p < .01; ***p < .001. *b* represents coefficients for models where variables are rescaled to range from 0-1 β represents coefficients for models where variables are standardized by standard deviation units (z-scored). For continuous variables, β coefficients represent the expected change in standard deviation units in the dependent variable per one standard deviation unit change in the respective independent variable. For categorical variables, β represents expected standard deviation change in the dependent variable if a member of the category.

**Table SK**

*Simple Slopes Analysis pmm Imputed Ideology2.0 Data*

|  | Ideology2.0 | | | | | |
| --- | --- | --- | --- | --- | --- | --- |
|  | Liberal participants  (Midpoint -1 SD) | | | Conservative participants  (Midpoint +1 SD) | | |
|  | b | SE | β | b | SE | β |
| Lower Cognitive Reflection (Mean -1 SD) | -.39* | .17 | -.37* | .15 | .17 | .14 |
| Higher Cognitive Reflection (Mean +1 SD) | -.42* | .17 | -.40* | .13 | .17 | .12 |

*Note:* Simple slopes analysis for the three-way interaction of interest for testing hypotheses 2-3. There is no evidence in support of either hypothesis 2 or hypothesis 3. **p* < .05. *b* represents coefficients for models where variables are rescaled to range from 0-1 β represents coefficients for models where variables are standardized by standard deviation units (z-scored). For continuous variables, β coefficients represent the expected change in standard deviation units in the dependent variable per one standard deviation unit change in the respective independent variable. For categorical variables, β represents expected standard deviation change in the dependent variable if a member of the category.

**Table SL**

*Fixed Effects from Main Effects Models in the Ideology2.0 Dataset Without Participant Random Effects*

|  | **Ideology2.0** | | | |
| --- | --- | --- | --- | --- |
|  | **Without Respondent Ideology** | | **With Respondent Ideology** | |
| **Variable** | b  (SE) | β  (SE) | b  (SE) | β  (SE) |
| Age | .03  (.015) | .01  (.007) | .03  (.015) | .01  (.007) |
| Ideological Difference | .32*  (.14) | .25*  (.11) | .32*  (.14) | .25*  (.11) |
| Education | .01  (.01) | .008  (.007) | .01  (.01) | .007  (.007) |
| Gender (1= Male) | .002  (.005) | .006  (.01) | .003  (.006) | .007  (.01) |
| Design (1= B) | .004  (.005) | .01  (.01) | .004  (.005) | .01  (.01) |
| White People v. Non-White People | .016  (.007) | .04  (.02) | .016  (.007) | .04  (.02) |
| Black People vs. Other Non-White People Except Whites | .001  (.01) | .004  (.03) | .001  (.01) | .004  (.03) |
| Hispanic People vs. Other Non-White People Except Blacks and Whites | -.02  (.01) | -.06  (.04) | -.02  (.01) | -.06  (.03) |
| **Cognitive Reflection** | -.003  (.02) | -.0001  (.01) | -.0009  (.01) | -.0007  (.01) |
| **Ideology of Respondent** | - | - | -.006  (.01) | -.005  (.01) |

*Note:* The dependent variable is the absolute value of the untransformed preference variable (-3 preference for liberal group, 3 preference for conservative group) recoded to range from 0-1. *p< .05, **p < .01; ***p < .001.*b* represents coefficients for models where variables are rescaled to range from 0-1 β represents coefficients for models where variables are standardized by standard deviation units (z-scored). For continuous variables, β coefficients represent the expected change in standard deviation units in the dependent variable per one standard deviation unit change in the respective independent variable. For categorical variables, β represents expected standard deviation change in the dependent variable if a member of the category.

**Table SM**

*Fixed Effects of Two-Way and Three-Way Interaction Models Ideology2.0 Without Participant Random Effects*

|  | **Two Way Interaction** | | **Three Way Interaction** | |
| --- | --- | --- | --- | --- |
| **Variable** | b  (SE) | β  (SE) | b  (SE) | β  (SE) |
| Age | -.01  (.02) | -.007  (.009) | -.01  (.02) | -.007  (.009) |
| Ideological Difference | .01  (.17) | -.11  (.16) | .01  (.17) | -.11  (.16) |
| Education | .0007  (.01) | .0006  (.009) | .0007  (.01) | .0006  (.009) |
| Gender (1= Male) | -.001  (.004) | -.004  (.01) | -.001  (.004) | -.004  (.01) |
| Design (1= B) | .001 (.003) | .004  (.01) | .001  (.003) | .004  (.01) |
| White People v. Non-White People | .008  (.004) | .03  (.02) | .008  (.005) | .03  (.02) |
| Black People vs. Other Non-White People Except Whites | .004  (.008) | .01  (.02) | .004  (.008) | .01  (.02) |
| Hispanic People vs. Other Non-White People Except Blacks and Whites | -.01  (.01) | -.04  (.03) | -.01  (.01) | -.04  (.04) |
| Cognitive Reflection | -.007  (.01) | -.008  (.01) | -.007  (.01) | -.007  (.01) |
| Ideology of Respondent | .32***  (.01) | .28***  (.01) | .32***  (.01) | .28***  (.01) |
| Cognitive Reflection*Ideology of Respondent | .003  (.03) | .001  (.007) | .003  (.03) | .005  (.007) |
| Cognitive Reflection*Ideological Difference | -.04  (.03) | -.01  (.01) | -.04  (.03) | -.01  (.007) |
| Ideological Difference*Ideology of Respondent | .97***  (.03) | .25***  (.01) | .97***  (.03) | .26***  (.01) |
| **Ideological Difference* Cognitive Reflection*Ideology of Participant** | **-** | **-** | **.02**  **(.06)** | **.002**  **(.005)** |

*Note:* The outcome variable is preference for liberal (0) or conservative (1) group in the pair. *p < .05, **p < .01; ***p < .001. *b* represents coefficients for models where variables are rescaled to range from 0-1 β represents coefficients for models where variables are standardized by standard deviation units (z-scored). For continuous variables, β coefficients represent the expected change in standard deviation units in the dependent variable per one standard deviation unit change in the respective independent variable. For categorical variables, β represents expected standard deviation change in the dependent variable if a member of the category.

**Table SN**

*Simple Slopes Analysis Without Participant Random Effects*

|  | Ideology2.0 | | | | | |
| --- | --- | --- | --- | --- | --- | --- |
|  | Liberal participants  (Midpoint -1 SD) | | | Conservative participants  (Midpoint +1 SD) | | |
|  | b | SE | β | b | SE | β |
| Lower Cognitive Reflection (Mean -1 SD) | -.39* | .17 | -.37* | .15 | .17 | .14 |
| Higher Cognitive Reflection (Mean +1 SD) | -.42* | .17 | -.40* | .13 | .17 | .12 |

*Note:* Simple slopes analysis for the three-way interaction of interest for testing hypotheses 2-3. There is no evidence in support of either hypothesis 2 or hypothesis 3. **p* < .05. *b* represents coefficients for models where variables are rescaled to range from 0-1 β represents coefficients for models where variables are standardized by standard deviation units (z-scored). For continuous variables, β coefficients represent the expected change in standard deviation units in the dependent variable per one standard deviation unit change in the respective independent variable.

**Tables SO**

*Group Specific Regression Results Ideology2.0 Dataset*

| Target: Black/White | b  (SE) |
| --- | --- |
| Intercept | .58***  (.06) |
| Ideology | .14  (.11) |
| Cognitive Reflection | .10  (.11) |
| Ideology*Cognitive Reflection | .05  (.20) |

| Target: Democrat/Republican | b  (SE) |
| --- | --- |
| Intercept | .43***  (.06) |
| Ideology | .91***  (.12) |
| Cognitive Reflection | .03  (.11) |
| Ideology*Cognitive Reflection | .01  (.20) |

| Target: Foreign/Local | b  (SE) |
| --- | --- |
| Intercept | .57***  (.14) |
| Ideology | .02  (.13) |
| Cognitive Reflection | .003  (.12) |
| Ideology*Cognitive Reflection | .003  (.22) |

| Target: Gay/Straight | b  (SE) |
| --- | --- |
| Intercept | .79***  (.07) |
| Ideology | .36***  (.13) |
| Cognitive Reflection | -.07  (.14) |
| Ideology*Cognitive Reflection | .10  (.23) |

| Target: Labor/Management | b  (SE) |
| --- | --- |
| Intercept | .51***  (.15) |
| Ideology | .02  (.11) |
| Cognitive Reflection | -.01  (.11) |
| Ideology*Cognitive Reflection | .008  (.20) |

| Target: Liberal/Conservative | b  (SE) |
| --- | --- |
| Intercept | .45***  (.06) |
| Ideology | .91***  (.11) |
| Cognitive Reflection | .01  (.11) |
| Ideology*Cognitive Reflection | .03  (.19) |

| Target: Mother/Father | b  (SE) |
| --- | --- |
| Intercept | .37***  (.07) |
| Ideology | -.04  (.13) |
| Cognitive Reflection | .06  (.12) |
| Ideology*Cognitive Reflection | -.01  (.21) |

| Target: Non-Profits/Corporations | b  (SE) |
| --- | --- |
| Intercept | .37***  (.14) |
| Ideology | .04  (.12) |
| Cognitive Reflection | .009  (.12) |
| Ideology*Cognitive Reflection | .03  (.23) |

| Target: Religious/Atheist | b  (SE) |
| --- | --- |
| Intercept | .65***  (.08) |
| Ideology | .43***  (.15) |
| Cognitive Reflection | -.07  (.15) |
| Ideology*Cognitive Reflection | .06  (.26) |

| Target: Science/Religion | b  (SE) |
| --- | --- |
| Intercept | .43***  (.08) |
| Ideology | .58***  (.14) |
| Cognitive Reflection | -.17  (.15) |
| Ideology*Cognitive Reflection | -.15  (.26) |

| Target: Socialism/Capitalism | b  (SE) |
| --- | --- |
| Intercept | .54***  (.15) |
| Ideology | .05  (.12) |
| Cognitive Reflection | .004  (.12) |
| Ideology*Cognitive Reflection | .04  (.22) |

*Note*: * p <.05, ** p <.01, *** p <.001

**Tables SP**

*Group Specific Regression Results Conservatives Ideology2.0*

| Target: Black/White | b  (SE) |
| --- | --- |
| Intercept | .64***  (.09) |
| Cognitive Reflection | .10  (.16) |

| Target: Democrat/Republican | b  (SE) |
| --- | --- |
| Intercept | .75***  (.10) |
| Cognitive Reflection | -.05  (.19) |

| Target: Foreign/Local | b  (SE) |
| --- | --- |
| Intercept | .56***  (.13) |
| Cognitive Reflection | -.004  (.19) |

| Target: Gay/Straight | b  (SE) |
| --- | --- |
| Intercept | .88***  (.09) |
| Cognitive Reflection | -.04  (.16) |

| Target: Labor/Management | b  (SE) |
| --- | --- |
| Intercept | .50***  (.12) |
| Cognitive Reflection | -.006  (.18) |

| Target: Liberal/Conservative | b  (SE) |
| --- | --- |
| Intercept | .76***  (.10) |
| Cognitive Reflection | -.08  (.14) |

| Target: Mother/Father | b  (SE) |
| --- | --- |
| Intercept | .36***  (.06) |
| Cognitive Reflection | .08  (.17) |

| Target: Non-Profits/Corporations | b  (SE) |
| --- | --- |
| Intercept | .34***  (.18) |
| Cognitive Reflection | -.0008  (.18) |

| Target: Religious/Atheist | b  (SE) |
| --- | --- |
| Intercept | .78***  (.08) |
| Cognitive Reflection | -.04  (.19) |

| Target: Science/Religion | b  (SE) |
| --- | --- |
| Intercept | .62***  (.08) |
| Cognitive Reflection | -.22  (.22) |

| Target: Socialism/Capitalism | b  (SE) |
| --- | --- |
| Intercept | .61***  (.17) |
| Cognitive Reflection | -.001  (.19) |

*Note*: * p <.05, ** p <.01, *** p <.001

**Supplemental Materials: Exploratory Analyses, Ideology2.0 Absolute Political Animosity/Favouritism Measures**

Absolute political animosity/favouritism measures in the dataset:

1. What are you gut feelings towards (target)?
   1. Likert scale ranging from 1- Strongly negative to 7 – Strongly positive
2. What are your actual feelings towards (target)?
   1. Likert scaling ranging from 1- Strongly negative to 7- Strongly positive
3. Considering only the negative things about (target) and ignoring the positive things, how negative are those things?
   1. Likert scale ranging from 1- Extremely negative to 6- Not at all negative
4. Considering only the positive things about (target) and ignoring the negative, how positive are those things?
   1. Likert scale ranging from 1- Not at all positive to 6- Extremely positive

**Model syntax no interaction models (without and with respondent ideology)**

lmerModList(attitude ~ crt + rating + educ + as.factor(gender) + Contrast1 + Contrast2 + Contrast3 + age + (1|target) + as.factor(measure) + (1|user_id), data = dat)

lmerModList(attitude ~ crt + rating + educ + politicalid + as.factor(gender) + Contrast1 + Contrast2 + Contrast3 + age + (1|target) + as.factor(measure) + (1|user_id), data = dat)

**Model syntax 2-way interaction model**

lmerModList(attitude ~ crt + rating + educ + politicalid + as.factor(gender) + Contrast1 + Contrast2 + Contrast3 + age + as.factor(measure) + rating*politicalid + crt*politicalid + crt*rating + (1|user_id) + (1|target) , data = dat)

**Model syntax 3-way interaction model**

lmerModList(attitude ~ crt + rating + educ + politicalid + as.factor(gender) + Contrast1 + Contrast2 + Contrast3 + age + as.factor(measure) + rating*crt*politicalid + (1|target) + (1|user_id), data = dat)

**Table SQ**

*Ideology2.0 Absolute Measure Main Effects Model Fixed Effects*

|  | **Without With**  **Respondent Respondent**  **Ideology Ideology** | | |
| --- | --- | --- | --- |
| **Variable** | b  (SE) | b  (SE) |  |
| Age | -.005  (.02) | -.005  (.02) |  |
| Ideological Difference | -.15  (.08) | -.15  (.08) |  |
| Education | .02  (.01) | .02  (.01) |  |
| Gender (1= Male) | -.0004  (.003) | -.0004  (.003) |  |
| White People v. Non-White People | -.001  (.003) | -.001  (.003) |  |
| Black People vs. Other Non-White People Except Whites | -.001  (.006) | -.001  (.006) |  |
| Hispanic People vs. Other Non-White People Except Blacks and Whites | .002  (.007) | .002  (.007) |  |
| Gut (1= gut measure) | .006  (.006) | .006  (.006) |  |
| Negative (1= neg measure) | -.17***  (.012) | -.17***  (.01) |  |
| Positive (1= positive measure) | .05***  (.01) | .05***  (.01) |  |
| **Cognitive Reflection** | .006  (.02) | .006  (.02) |  |
| **Ideology of Respondent** | - | .0002  (.008) |  |

*Note:* The dependent variable is the absolute value of the untransformed preference variable (-3 preference for liberal group, 3 preference for conservative group) recoded to range from 0-1. *p< .05, **p < .01; ***p < .001. *b* represents coefficients for models where variables are rescaled to range from 0-1.

**Table SR**

*Fixed Effects of Two-Way and Three-Way Interaction Models Ideology2.0 Absolute Measures*

|  | **Two Way Three**  **Interaction Way**  **Interaction** | |
| --- | --- | --- |
| **Variable** | b  (SE) | b  (SE) |
| Age | -.005  (.02) | -.005  (.02) |
| Ideological Difference | -.04  (.08) | -.04  (.08) |
| Education | .02  (.01) | .02  (.01) |
| Gender (1= Male) | -.004  (.003) | -.0004  (.003) |
| White People v. Non-White People | -.001  (.003) | -.001  (.003) |
| Black People vs. Other Non-White People Except Whites | -.001  (.006) | -.001  (.006) |
| Hispanic People vs. Other Non-White People Except Blacks and Whites | .002  (.006) | .002  (.006) |
| Gut (1= gut measure) | .006  (.006) | .006  (.006) |
| Negative (1= Negative measure) | -.17  (.01) | -.17  (.01) |
| Positive (1 = Positive measure) | .05***  (.009) | .05***  (.01) |
| Cognitive Reflection | .006  (.02) | .006  (.02) |
| Ideology of Respondent | .0002  (.008) | .0002  (.008) |
| Cognitive Reflection*Ideology of Respondent | .0006  (.01) | .0006  (.02) |
| Cognitive Reflection*Ideological Difference | -.06  (.05) | -.06  (.05) |
| Ideological Difference*Ideology of Respondent | .85***  (.04) | .85***  (.04) |
| **Ideological Difference* Cognitive Reflection*Ideology of Participant** | **-** | **.003**  **(.07)** |

*Note:* The outcome variable is preference for liberal (0) or conservative (1) group in the pair. *p < .05, **p < .01; ***p < .001. *b* represents coefficients for models where variables are rescaled to range from 0-1.

**Table SS**

*Ideology2.0 Results Main Effects Model Fixed Effects Without Demographic Controls*

|  | **Ideology2.0** | | | |
| --- | --- | --- | --- | --- |
|  | **Without Respondent Ideology** | | **With Respondent Ideology** | |
| **Variable** | b  (SE) | β  (SE) | b  (SE) | β  (SE) |
| **Ideological Difference** | .32  (.14) | .25  (.11) | .32  (.14) | .25  (.11) |
| **Cognitive Reflection** | .005  (.05) | .003  (.01) | .001  (.01) | .001  (.01) |
| **Ideology of Respondent** | - | - | -.006  (.01) | -.004  (.01) |

*Note:* The dependent variable is the absolute value of the untransformed preference variable (-3 preference for liberal group, 3 preference for conservative group) recoded to range from 0-1. *p< .05, **p < .01; ***p < .001. *b* represents coefficients for models where variables are rescaled to range from 0-1 β represents coefficients for models where variables are standardized by standard deviation units (z-scored). For continuous variables, β coefficients represent the expected change in standard deviation units in the dependent variable per one standard deviation unit change in the respective independent variable.

**Table ST**

*Fixed Effects of Two-Way and Three-Way Interaction Models Ideology2.0 No Controls*

|  | **Two Way Interaction** | | **Three Way Interaction** | |
| --- | --- | --- | --- | --- |
| **Variable** | b  (SE) | β  (SE) | b  (SE) | β  (SE) |
| Ideological Difference | .01  (.17) | -.11  (.16) | .01  (.17) | -.11  (.16) |
| Cognitive Reflection | -.007  (.01) | -.007  (.01) | -.007  (.01) | -.007  (.01) |
| Ideology of Respondent | .32***  (.01) | .28***  (.01) | .32***  (.02) | .28***  (.02) |
| Cognitive Reflection*Ideology of Respondent | .003  (.03) | .006  (.007) | .003  (.03) | .008  (.007) |
| Cognitive Reflection*Ideological Difference | -.04  (.03) | -.01  (.008) | -.04  (.03) | -.01  (.007) |
| Ideological Difference*Ideology of Respondent | .97***  (.03) | .25***  (.01) | .97***  (.03) | .25***  (.01) |
| **Ideological Difference* Cognitive Reflection*Ideology of Participant** | **-** | **-** | **.02**  **(.07)** | **.002**  **(.005)** |

*Note:* The outcome variable is preference for liberal (0) or conservative (1) group in the pair. *p < .05, **p < .01; ***p < .001. *b* represents coefficients for models where variables are rescaled to range from 0-1 β represents coefficients for models where variables are standardized by standard deviation units (z-scored). For continuous variables, β coefficients represent the expected change in standard deviation units in the dependent variable per one standard deviation unit change in the respective independent variable.

**Table SU**

*Simple Slopes Analysis No Control Variables Ideology2.0 Data*

|  | Ideology2.0 | | | | | |
| --- | --- | --- | --- | --- | --- | --- |
|  | Liberal participants  (Midpoint -1 SD) | | | Conservative participants  (Midpoint +1 SD) | | |
|  | B | SE | β | b | SE | β |
| Lower Cognitive Reflection (Mean -1 SD) | -.38* | .17 | -.35* | .17 | .17 | .15 |
| Higher Cognitive Reflection (Mean +1 SD) | -.41* | .17 | -.38* | .14 | .17 | .13 |

*Note:* Simple slopes analysis for the three-way interaction of interest for testing hypotheses 2-3. There is no evidence in support of either hypothesis 2 or hypothesis 3. **p* < .05. *b* represents coefficients for models where variables are rescaled to range from 0-1 β represents coefficients for models where variables are standardized by standard deviation units (z-scored). For continuous variables, β coefficients represent the expected change in standard deviation units in the dependent variable per one standard deviation unit change in the respective independent variable.

**Table SV: Correlation Between Cognitive Reflection and Conservatism Ideology2.0 dataset**

|  |
| --- |
| -.12 |
